# Supplementary material for: Integrating pharmacogenomics in three Middle Eastern countries’ healthcare (Lebanon, Qatar, and Saudi Arabia): Current insights, challenges, and strategic directions
Source: PLoS One. 2025 Apr 11;20(4):e0319042. doi: 10.1371/journal.pone.0319042 (PMC11991729; doi:10.1371/journal.pone.0319042)
Supplement: S1 Table — Categorical variables are shown as numbers (n) and percentages (%). n: sample size. A χ2 test of independence was used to test if the differences between Lebanon and Qatar are significantly different. (DOCX) [file pone.0319042.s001.docx]

S1 Table: Interest level of the participants according to the age groups.

|  |  | **Interest level** | | | |  |  |
| --- | --- | --- | --- | --- | --- | --- | --- |
| **Age categories** |  | Not Interested | Slightly Interested | Interested | Very Interested | **Chi-squared** | **P** |
| **18-25 (years)** | Count | 3 | 17 | 38 | 35 | 14 | 0.254 |
|  | % | 100.0% | 51.5% | 44.2% | 49.3% |  |  |
| **26-35 (years)** | Count | 0 | 6 | 19 | 15 |  |  |
|  | % |  | 18.2% | 22.1% | 21.1% |  |  |
| **36-45 (years)** | Count | 0 | 5 | 21 | 18 |  |  |
|  | % |  | 15.2% | 24.4% | 25.4% |  |  |
| **46-55 (years)** | Count | 0 | 2 | 7 | 3 |  |  |
|  | % |  | 6.1% | 8.1% | 4.2% |  |  |
| **> 55 (years)** | Count | 0 | 3 | 1 | 0 |  |  |
|  | % |  | 9.1% | 1.2% | 0.0% |  |  |
|  | Count | 3 | 33 | 86 | 71 |  |  |

Categorical variables are shown as numbers (n) and percentages (%). n: sample size. A χ2 test of independence was used to test if the differences between Lebanon and water are significantly different.
