# Supplementary material for: Integrating pharmacogenomics in three Middle Eastern countries’ healthcare (Lebanon, Qatar, and Saudi Arabia): Current insights, challenges, and strategic directions
Source: PLoS One. 2025 Apr 11;20(4):e0319042. doi: 10.1371/journal.pone.0319042 (PMC11991729; doi:10.1371/journal.pone.0319042)
Supplement: Attachment — Submitted filename: PONE-D-24-33568_R1 (1).pdf [file pone.0319042.s005.pdf]

PLOS ONE

Integrating Pharmacogenomics in Three Middle Eastern Countries' Healthcare (Lebanon, Qatar, and Saudi Arabia): Current Insights, Challenges, and Strategic Directions

--Manuscript Draft--

Manuscript Number:	PONE-D-24-33568R1
Article Type:	Research Article
Full Title:	Integrating Pharmacogenomics in Three Middle Eastern Countries' Healthcare (Lebanon, Qatar, and Saudi Arabia): Current Insights, Challenges, and Strategic Directions
Short Title:	Pharmacogenomics in three Middle Eastern Countries' Healthcare: Insights, Challenges, and Strategic Directions
Corresponding Author:	Rajaa Fakhoury<br>Beirut Arab University<br>Beirut , LEBANON
Keywords:	Pharmacogenomics; Lebanon; Qatar; Saudi Arabia; precision medicine.
Abstract:	Background and Objectives: Pharmacogenomics (PGx) leverages genomic information to tailor drug therapies, enhancing precision medicine. Despite global advancements, its implementation in Lebanon, Qatar, and Saudi Arabia faces unique challenges in clinical integration. This study aimed to investigate PGx attitudes, knowledge implementation, associated challenges, forecast future educational needs, and compare findings across the three countries.<br><br>Methods: This cross-sectional study utilized an anonymous, self-administered online survey distributed to healthcare professionals, academics, and clinicians in Lebanon, Qatar, and Saudi Arabia. The survey comprised 18 questions to assess participants' familiarity with PGx, current implementation practices, perceived obstacles, potential integration strategies, and future educational needs.<br><br>Results: The survey yielded 337 responses from healthcare professionals across the three countries. Data revealed significant variations in PGx familiarity and educational involvement. Qatar and Saudi Arabia participants were more familiar with PGx compared to Lebanon (83%, 75%, and 67%, respectively). Participation in PGx-related talks was most prevalent in Saudi Arabia (96%), followed by Qatar (53%) and Lebanon (35%). Key challenges identified included test cost and reimbursement, insufficient physician knowledge, and lack of infrastructure. Lebanon reported the highest concern for test costs (16%), compared to the lowest in Saudi Arabia (5%). Despite these challenges, a strong consensus emerged on PGx's potential to improve patient outcomes, with over 86% of respondents in all three countries expressing this belief. Educational interest areas varied by country, with strong interest in PGx for cancer chemotherapy in Saudi Arabia and Lebanon and for diabetes mellitus in Qatar.<br><br>Conclusion: This study highlights the significant influence of varied educational backgrounds and infrastructural limitations on PGx implementation across Lebanon, Qatar, and Saudi Arabia. The findings emphasize the need for targeted strategies in each country to address these distinct barriers. Integrating PGx education into healthcare training programs and clinical workflows could unlock PGx's potential to optimize patient care.
Order of Authors:	Said El Shamieh, PhD.
	Rimah Abdullah Saleem
	Dalal Hammoudi Halat
	Hana M A Fakhoury
	Kholoud Bastaki
	Mirna Fawaz

Powered by Editorial Manager® and ProduXion Manager® from Aries Systems Corporation

	Ahmed Melki
	Rajaa Fakhoury
**Response to Reviewers:**	Reviewer #1:<br><br>1. Do you have the data on the level of education for the subjects? (eg. PhD, Masters, Bsc)<br>would be interesting to see if there is a difference in acceptance towards Pgx between each group<br><br>We thank the reviewer for the pertinent comment. Accordingly, we have added all our participants' academic degrees (Table 1, page 20). We have described these results (lines 177-182, page 8) and then compared the difference in acceptance towards PGx in each group (Table 4, page 23).<br>This analysis showed interesting patterns that we describe in the Results section (lines 247-261, page 10 and lines 262-263, page 11) and the Discussion section (line 336-342, page 14).<br><br>2. Would be great if you also have data on their awareness of any guidelines related to Pgx.<br><br>We thank the reviewer for this important comment. Unfortunately, we don't have this data and we have added this to the limitations section (line 357-359, page 14)<br><br>3. Could you elaborate more on the level of interest (for groups who are interested to know more) with age?<br><br>We thank the reviewer for highlighting this issue. We analyzed the interest level according to the different age groups and found no significant associations (Supplementary Table). We also describe this in the results section (lines 241-242, page 10).<br><br>4. Is there data on pgx practice in Lebanon, Qatar, and Saudi Arabia, and does it collaborate with the level of interest for each respondent grouped by countries?<br><br>We thank the reviewer for this comment. Data on pharmacogenomics practice in the three countries exists to some extent in literature, and this was highlighted in the Introduction section with relevant references: references 16-27 for Lebanon, 29-36 for Qatar, and 37-40 for Saudi Arabia. Yes, the level of interest varied across the three countries, as shown in Table 2 (page 21). We have described all the details in the results section (lines 186-196, page 8).<br><br>Reviewer #2: Reviewer Comments:<br><br>1. Title Appropriateness: The title of the manuscript is not suitable as it refers to the Middle East, which comprises 18 countries. However, the study is limited to three countries: Lebanon, Qatar, and Saudi Arabia. The data presented may not adequately represent the healthcare population of the entire region. I recommend revising the title to "Integrating Pharmacogenomics in Three Middle Eastern Countries' Healthcare (Lebanon, Qatar, and Saudi Arabia): Current Insights, Challenges, and Strategic Direction" to more accurately reflect the scope of the research.<br><br>We thank the reviewer for pointing out this issue. We have used the reviewer's suggestion for the title of our article.<br><br>2. Data Sufficiency: The results and areas of coverage presented in the manuscript are insufficient for publication in a Q1 journal. To enhance the study's contribution to the field, I suggest providing a broader dataset or deeper insights into the healthcare practices and pharmacogenomics landscape within the selected countries.

Powered by Editorial Manager® and ProduXion Manager® from Aries Systems Corporation

We thank the reviewer for being keen about data coverage presented in the manuscript. Indeed, a broader dataset would be beneficial suggestion, and providing it would be interesting for ongoing research. For the current study, we exhausted different means of contacting pharmacogenomics experts in the three countries, by approaching them personally, given their known expertise in the field and their reputation. We also sought their support in disseminating the survey among their teams. We reached to broader pharmacist communities to seek specialist participation. Given that the primary authors from each country are academicians, we used our contacts among specialized academicians and researchers to get more engagement in the survey. However, we need to appreciate the relative novelty of the field in the region and the fact that Qatar and Lebanon are both small countries. Finding specialists within the pharmacogenomics domain whose participation adds value to the study should be endeavored carefully. Nevertheless, we have accounted for sample size as one of the study limitations in the Discussion section.
To satisfy the reviewer's concerns and enhance the study contribution to the field, we provided deeper insights into our data by additional analysis on academic degrees, interests in pharmacogenomics, and age. The analysis according to the academic degree showed significantly different patterns as the interest for PGX varied depending on the academic degree (PhD vs. MD vs. BS in Pharmacy vs. BS in a healthcare field (Results, lines 247-261, page 10 and lines 262-263, page 11, and Table 4, page 23). In contrast, the age of the participants was not associated with the interest levels (lines 241-242, page 10).

3. Clarification of Roles: The roles of academics, healthcare professionals, and clinicians appear to overlap without clear distinctions. The manuscript should specify the areas of specialty for the academic participants, as both clinicians and healthcare professionals may also serve as academicians. Clarifying these roles will strengthen the manuscript's clarity and precision.

We thank the reviewer for pointing out this pertinent issue. After carefully checking the clinicians enrolled in our study, we noticed that only two (from Lebanon) had teaching activities. The latter clinicians specialized in Pharmacogenomics/Personalized Medicine and Medical Genetics. Interestingly, our academics were all those of these two specialties. As this is a critical point, we have now clarified it in the Discussion section (page 14, line 344-348).

4. Statistical Analysis Issues: The statistical analysis presented is inadequate. For example, the statement, “A $\chi 2$ test of independence was used to test if the differences between Lebanon and water are significantly different,” is confusing and lacks clarity. It does not provide a significance value for the measurable variables, which is essential for interpreting the results. I recommend revising this section to include clear explanations of the statistical methods used and the significance of the findings.

We do entirely agree with the reviewer and acknowledge that we didn’t provide initially enough details concerning the statistical analysis section. Accordingly, we have provided all the necessary details in the methods section (lines 142-157, page 6).

5. Overlap in Pharmacogenomic Specifications: There are overlapping specifications regarding pharmacogenomics topics. For instance, conditions such as diabetes mellitus and cardiovascular diseases may be influenced by a combination of genetic, environmental, and lifestyle factors, which are similar to the characteristics of complex diseases. It is important to delineate these topics clearly to avoid confusion and enhance the manuscript’s focus.

We agree with the reviewer’s observation regarding the overlap in some pharmacogenomics topics. In response, we emphasize that including these overlapping categories was a deliberate decision to reflect our respondents' varied interests and areas of expertise. Although these conditions share a common etiology, their pharmacogenomic implications and therapeutic approaches are distinct. For instance, internal medicine and family medicine practitioners typically manage complex

	diseases. In contrast, endocrinologists predominantly focus on endocrine disorders like diabetes mellitus, while cardiologists specialize in cardiovascular diseases. By delineating these categories, we captured clinicians' variable interest in PGx topics. We have now discussed this point (lines 318-325, page 13).<br><br>6. Discussion Quality: The discussion section is well-written and effectively outlines the challenges faced in the integration of pharmacogenomics, along with proposed strategies to overcome these obstacles. This part of the manuscript is commendable and adds significant value to the overall narrative.<br><br>We thank the reviewers for the interest they showed in recommending our discussion section and highlighting its value.<br><br>Based on the above, we hope to have satisfied the reviewers' comments and to have the amended version of the manuscript acceptable for publication in PLOS One.
Additional Information:	
**Question**	**Response**
**Financial Disclosure**<br><br>Enter a financial disclosure statement that describes the sources of funding for the work included in this submission. Review the submission guidelines for detailed requirements. View published research articles from *PLOS ONE* for specific examples.<br><br>This statement is required for submission and **will appear in the published article** if the submission is accepted. Please make sure it is accurate.<br><br>**Funded studies**<br>Enter a statement with the following details:<br>• Initials of the authors who received each award<br>• Grant numbers awarded to each author<br>• The full name of each funder<br>• URL of each funder website<br>• Did the sponsors or funders play any role in the study design, data collection and analysis, decision to publish, or preparation of the manuscript?<br><br>Did you receive funding for this work?	The author(s) received no specific funding for this work.
**Competing Interests**<br><br>Use the instructions below to enter a competing interest statement for this	The authors have declared that no competing interests exist.

submission. On behalf of all authors, disclose any competing interests that could be perceived to bias this work—acknowledging all financial support and any other relevant financial or non-financial competing interests.<br><br>This statement is required for submission and **will appear in the published article** if the submission is accepted. Please make sure it is accurate and that any funding sources listed in your Funding Information later in the submission form are also declared in your Financial Disclosure statement.<br><br>View published research articles from *PLOS ONE* for specific examples.<br><br>**NO authors have competing interests**<br><br>Enter: *The authors have declared that no competing interests exist.*<br><br>**Authors with competing interests**<br><br>Enter competing interest details beginning with this statement:<br><br>*I have read the journal's policy and the authors of this manuscript have the following competing interests: [insert competing interests here]*<br><br>* typeset	
**Ethics Statement**<br><br>Enter an ethics statement for this submission. This statement is required if the study involved:<br><br>• Human participants<br>• Human specimens or tissue<br>• Vertebrate animals or cephalopods<br>• Vertebrate embryos or tissues<br>• Field research<br><br>Write "N/A" if the submission does not	Ethical approval for this study was obtained from the institutional review boards of the participating universities: Beirut Arab University (2023-H-0153-HS-R-0545), Qatar University (QU-IRB 1995-E/23), and Alfaisal University (IRB-20270). Informed consent was obtained from all participants online, ensuring their confidentiality and the right to withdraw from the study without any consequences. Participants were informed that all collected data would be anonymous and confidential, with only the principal investigator having access to the data. Completing and submitting the survey was considered an agreement to participate.

require an ethics statement.<br><br>General guidance is provided below. Consult the submission guidelines for detailed instructions. **Make sure that all information entered here is included in the Methods section of the manuscript.**<br><br>**Format for specific study types**<br><br>**Human Subject Research (involving human participants and/or tissue)**<br>• Give the name of the institutional review board or ethics committee that approved the study<br>• Include the approval number and/or a statement indicating approval of this research<br>• Indicate the form of consent obtained (written/oral) or the reason that consent was not obtained (e.g. the data were analyzed anonymously)<br><br>**Animal Research (involving vertebrate animals, embryos or tissues)**<br>• Provide the name of the Institutional Animal Care and Use Committee (IACUC) or other relevant ethics board that reviewed the study protocol, and indicate whether they approved this research or granted a formal waiver of ethical approval<br>• Include an approval number if one was obtained<br>• If the study involved *non-human primates*, add *additional details* about animal welfare and steps taken to ameliorate suffering<br>• If anesthesia, euthanasia, or any kind of animal sacrifice is part of the study, include briefly which substances and/or methods were applied<br><br>**Field Research**<br><br>Include the following details if this study involves the collection of plant, animal, or other materials from a natural setting:<br>• Field permit number<br>• Name of the institution or relevant body that granted permission	
**Data Availability**	Yes - all data are fully available without restriction

Authors are required to make all data underlying the findings described fully available, without restriction, and from the time of publication. PLOS allows rare exceptions to address legal and ethical concerns. See the PLOS Data Policy and FAQ for detailed information.<br><br>A Data Availability Statement describing where the data can be found is required at submission. Your answers to this question constitute the Data Availability Statement and **will be published in the article**, if accepted.<br><br>**Important:** Stating 'data available on request from the author' is not sufficient. If your data are only available upon request, select 'No' for the first question and explain your exceptional situation in the text box.<br><br>Do the authors confirm that all data underlying the findings described in their manuscript are fully available without restriction?	
**Describe where the data may be found in full sentences. If you are copying our sample text, replace any instances of XXX with the appropriate details.**<br><br>• If the data are **held or will be held in a public repository**, include URLs, accession numbers or DOIs. If this information will only be available after acceptance, indicate this by ticking the box below. For example: *All XXX files are available from the XXX database (accession number(s) XXX, XXX.*).<br>• If the data are all contained **within the manuscript and/or Supporting Information files**, enter the following: *All relevant data are within the manuscript and its Supporting Information files.*<br>• If neither of these applies but you are able to provide **details of access elsewhere**, with or without limitations, please do so. For example:	All relevant data are within the manuscript and its Supporting Information files.

Data cannot be shared publicly because of [XXX]. Data are available from the XXX Institutional Data Access / Ethics Committee (contact via XXX) for researchers who meet the criteria for access to confidential data.<br><br>*The data underlying the results presented in the study are available from (include the name of the third party and contact information or URL).*<br>• This text is appropriate if the data are owned by a third party and authors do not have permission to share the data.<br><br>* typeset	
Additional data availability information:	Tick here if the URLs/accession numbers/DOIs will be available only after acceptance of the manuscript for publication so that we can ensure their inclusion before publication.

Manuscript

Click here to access/download;Manuscript;PGx in Lebanon Qatar and KSA_REVISED No track.docx

Integrating Pharmacogenomics in Three Middle Eastern Countries' Healthcare (Lebanon, Qatar, and Saudi Arabia): Current Insights, Challenges, and Strategic Directions

Said El Shamieh[1], Rimah Abdullah Saleem[2], Dalal Hammoudi Halat[3], Hana M A Fakhoury[2], Kholoud Bastaki[4], Mirna Fawaz[5], Ahmed Malki[3], Rajaa Fakhoury[1,2 #].

[*] Contributed equally

[1] Department of Medical Laboratory Technology, Faculty of Health Sciences, Beirut Arab University, Beirut 11-5020, Lebanon.

[2] Department of Biochemistry and Molecular Medicine, College of Medicine, Alfaisal University, Riyadh, Saudi Arabia.

[3] Academic Quality Department, QU Health, Qatar University, P.O. Box 2713, Doha, Qatar.

[4] Clinical and Pharmacy Practice Department, College of Pharmacy, QU Health, Qatar University, Doha, Qatar.

[5] Department of Nursing, Faculty of Health Sciences, Beirut Arab University, Beirut 11-5020, Lebanon.

[#] Corresponding author: Rajaa Fakhoury rfakhoury@bau.edu.lb

Abstract

Background and Objectives: Pharmacogenomics (PGx) leverages genomic information to tailor drug therapies, enhancing precision medicine. Despite global advancements, its implementation in Lebanon, Qatar, and Saudi Arabia faces unique challenges in clinical integration. This study aimed to investigate PGx attitudes, knowledge implementation, associated challenges, forecast future educational needs, and compare findings across the three countries.

Methods: This cross-sectional study utilized an anonymous, self-administered online survey distributed to healthcare professionals, academics, and clinicians in Lebanon, Qatar, and Saudi Arabia. The survey comprised 18 questions to assess participants' familiarity with PGx, current implementation practices, perceived obstacles, potential integration strategies, and future educational needs.

Results: The survey yielded 337 responses from healthcare professionals across the three countries. Data revealed significant variations in PGx familiarity and educational involvement. Qatar and Saudi Arabia participants were more familiar with PGx compared to Lebanon (83%, 75%, and 67%, respectively). Participation in PGx-related talks was most prevalent in Saudi Arabia (96%), followed by Qatar (53%) and Lebanon (35%). Key challenges identified included test cost and reimbursement, insufficient physician knowledge, and lack of infrastructure. Lebanon reported the highest concern for test costs (16%), compared to the lowest in Saudi Arabia (5%). Despite these challenges, a strong consensus emerged on PGx's potential to improve patient outcomes, with over 86% of respondents in all three countries expressing this belief. Educational interest areas varied by country, with strong interest in PGx for cancer chemotherapy in Saudi Arabia and Lebanon and for diabetes mellitus in Qatar.

Conclusion: This study highlights the significant influence of varied educational backgrounds and infrastructural limitations on PGx implementation across Lebanon, Qatar, and Saudi Arabia. The findings emphasize the need for targeted strategies in each country to address these distinct barriers. Integrating PGx education into healthcare training programs and clinical workflows could unlock PGx's potential to optimize patient care.

Keywords: Pharmacogenomics, Lebanon, Qatar, Saudi Arabia, precision medicine.

Introduction

The completion of the human genome sequencing project in 2001 marked a pivotal moment in biomedical research, laying the foundation for the field of pharmacogenomics (PGx) (1). PGx examines the influence of an individual's genetic makeup on their response to drugs, using DNA signatures to guide treatment and inform drug development and testing (2). Recent advances in PGx have deepened our understanding of the factors contributing to individual variations in drug response. Genetic variants correlated with drug responses can now be utilized to tailor drug selection and dosing for each patient (3). As a cornerstone of precision medicine, PGx integrates genomic, environmental, and lifestyle information to inform medical management decisions, providing a more precise approach to disease prevention, diagnosis, and treatment (4-6). The increasing number of US FDA approvals for precision therapeutics underscores the growing impact of PGx (7). However, several challenges impede its routine implementation, including modifications of current clinical practices, knowledge gaps, a lack of interest among healthcare professionals, technological hurdles, and cost-effectiveness issues, particularly in developing countries (8-11).

The literature highlights a disparity in PGx research and implementation between low-resource settings and developed economies, each facing unique obstacles and needs (12, 13). There is a significant need for education and training among healthcare providers to effectively interpret and apply PGx data in clinical settings. This includes integrating PGx into medical curricula and continuous professional development programs (14, 15). In Lebanon, efforts in PGx have been progressing, with several pharmacogenetics laboratories established in the past decade (16). Studies employing next-generation sequencing and other genotyping techniques demonstrate active participation in this field (17-23). However, an international survey of PGx experts, including Lebanon, indicated that clinical implementation remains challenging despite the availability of PGx tests and a positive attitude towards their applications. Moreover, while PGx education is included in the curricula of Lebanese higher education institutions (24, 25), there is still a need for further education to improve foundational knowledge and capitalize on significant opportunities for advancement (26-28).

In contrast, Qatar has made considerable strides in PGx, supported by an advanced healthcare infrastructure, a national genome program, and numerous educational initiatives focused on precision medicine (29). Research in Qatar encompasses a variety of pharmacogenomic applications, including antipsychotics, antidepressants, immunosuppressants, warfarin, vitamin D deficiency, and type 2 diabetes

risk stratification. To support PGx implementation, Qatar has undertaken numerous educational initiatives, including courses for healthcare providers, conferences, curricular updates, and the establishment of precision medicine committees at institutional and national levels (29-35). To support PGx implementation, Qatar has undertaken numerous educational initiatives, including courses for healthcare providers, conferences, curricular updates, and the establishment of precision medicine committees at institutional and national levels (29). Studies indicate that physicians and pharmacists in Qatar possess positive attitudes and are motivated to engage in PGx-related clinical applications and patient education (36).

PGx is an emerging field in Saudi Arabia with significant implications for personalized medicine. However, there is a notable lack of knowledge and understanding of PGx among healthcare professionals, including community pharmacists and physicians. (37). Research shows that many individuals in the local population carry PGx variants, which affect a substantial percentage of medications dispensed annually, highlighting the need for tailored prescription practices (38-40).

Despite the rapid growth of PGx literature and increasing support from regulatory agencies, there remains a gap in the literature regarding its actual status and implementation (41). Enhancing education and training initiatives to include pharmacogenomics, improving healthcare professionals' expertise, and raising awareness among policymakers about the importance of PGx could leverage future insights for better healthcare applications (42). Focused exploration of PGx attitudes, knowledge, implementation, and associated barriers and needs in Lebanon, Qatar, and Saudi Arabia would be instrumental in unlocking PGx's full potential in improving patient care and healthcare systems. To our knowledge, a consolidated investigation of these PGx pillars from the perspective of healthcare professionals and specialists has not been previously undertaken. This study aims to examine the status of PGx implementation in Lebanon, Qatar, and Saudi Arabia, identify associated challenges, predict future educational needs, and compare findings across these three countries.

Methodology

Ethical statement and informed consent

Ethical approval for this study was obtained from the institutional review boards of the participating universities: Beirut Arab University (2023-H-0153-HS-R-0545), Qatar University (QU-IRB 1995-E/23), and Alfaisal University (IRB-20270). Informed consent was obtained from all participants online, ensuring their confidentiality and the right to withdraw from the study without any consequences. Participants were informed that all collected data would be anonymous and confidential, with only the principal investigator having access to the data. Completing and submitting the survey was considered an agreement to participate.

Study design

This study utilized a quantitative cross-sectional research design, involving healthcare professionals (pharmacists, nurses, medical laboratory technologists), university academics, and clinicians from Lebanon, Qatar, and Saudi Arabia. Data was collected through a voluntary, anonymous, private survey to gather PGx perspectives from specialists in the three countries. The study was conducted and reported following the Strengthening the Reporting of Observational Studies in Epidemiology (STROBE) guidelines (43).

Survey instrument

The survey questions were adapted from the previously published manuscript by of Ghaddar et al. , with the approval of Pr. Nathalie Zgheib (44). The instrument comprised 18 questions divided into several sections. The first section collected demographic information such as age, gender, country of residence, occupation, and years of experience in clinical practice or research. The second section focused on PGx implementation, examining participants' familiarity with the concept, attendance or presentation of educational talks, and integration of pharmacogenetic testing into their practice or research. The third section explored the obstacles faced in implementing PGx, asking participants to identify the top five obstacles from a provided list. The final section inquired about future considerations, including measures to overcome barriers in PGx implementation, belief in the potential of PGx to improve patient outcomes, interest in learning about pharmacogenomics, and specific topics of interest.

Before launching, the survey was pilot-tested with a small group of healthcare professionals to ensure content clarity and relevance. Based on the pilot test, the survey questions were modified, added, or deleted to improve the content. The pilot responses were not included in the final analysis. The survey items were tailored to country-specific practice norms through consultation with experts from Lebanon and Qatar, where data collection began. For instance, the PGx of pain management was added due to its common testing in Qatari patients.

Data collection

Participants were recruited through purposive sampling, targeting professionals directly or indirectly involved in PGx. PGx opinion leaders or experts in each country were approached personally via direct email or social media and asked to complete and disseminate the survey within their teams. The survey was launched on September 27th, 2023, and data collection ended on December 23rd, 2023, in Lebanon; January 24th, 2024, in Qatar; and June 7th, 2024, in Saudi Arabia. Participants were briefed about the study's aim in the survey's introductory paragraph and directed to respond anonymously through Google Forms. The survey took approximately 4 minutes to complete.

Statistical analysis

All statistical analyses were performed using SPSS software (version 26; SPSS Inc., Chicago, IL). Descriptive statistics were used to summarize demographic characteristics and survey responses, with categorical variables expressed as frequencies and percentages. Group comparisons were conducted using the chi-square (χ^2) test of independence to assess whether there were significant differences across the study groups; (1) countries, (2) participants' jobs, (3) academic degrees. The measurable variables included demographic characteristics such as age categories, gender, professional roles, and years of professional experience. Familiarity and involvement with PGx were also compared across the study groups, including familiarity with the concept of PGx, attendance or presentation of educational talks on PGx in the past two years, and the integration of PGx into clinical or research practices. Additionally, future interest and educational needs were assessed by comparing beliefs in PGx's potential to improve patient outcomes, levels of interest in learning about PGx, and specific PGx topics of interest. For each measurable variable, contingency tables were created to cross-tabulate responses by study group, and the χ^2 test was applied to assess independence between these variables. The statistical significance threshold was set at $P \leq 0.05$. When expected cell counts in the contingency table were less than 5, Fisher's exact test was used instead

to ensure the validity of the results. Results are reported with the corresponding χ^2, and P-values to provide transparency and context for the observed differences.

Results

The demographic characteristics of the study participants from Lebanon, Qatar, and Saudi Arabia are detailed in Table 1. The total sample comprised 337 participants: 145 (43%) from Lebanon, 137 (41%) from Qatar, and 55 (16%) from Saudi Arabia. Age distribution showed a significant skew, with a larger proportion of younger participants (18-25 years) in Lebanon (66%) compared to Qatar (4.5%) and Saudi Arabia (4%). Middle-aged groups (26-45 years) were more prevalent in Qatar, while 57% of respondents from Saudi Arabia were between 36 and 45 years old. Gender distribution was similar across the countries, with females constituting the majority: 66% in Lebanon, 70% in Qatar, and 51% in Saudi Arabia. Academics and clinicians were similarly distributed between Qatar and Lebanon. However, there was a notable disparity in healthcare professionals, with Lebanon having a slightly higher proportion (71% vs. 66% in Qatar, Table 1). In terms of academic degrees, the PhD holders represented 30% of our participants, primarily in Saudi Arabia (69%), with Qatar (25%) and Lebanon (20%) trailing. MD holders were 9%, with distributions of 13% in Saudi Arabia, 9% in Qatar, and 7% in Lebanon. BS in Pharmacy holders made up 19%, dominated by Qatar (33%), followed by Saudi Arabia (18%) and Lebanon (6%). The Bachelor's degree (healthcare professionals), the largest group at 42%, were concentrated in Lebanon (67%) and Qatar (33%), with none in Saudi Arabia. Professional experience varied, with Qatar and Saudi Arabia showing higher representation in the more experienced categories than Lebanon (11-20 years and more than 20 years).

The perspectives on PGx across the three countries regarding beliefs, educational interests, and preferred topics were also analyzed (Table 2). Participants from all three countries agreed on the potential of PGx to improve patient outcomes, with no significant differences across the three study groups. Interest in learning about PGx varied significantly among the regions ($P<0.005$, Table 2). Saudi Arabia recorded the highest proportion of participants very interested in PGx (55%), compared to Lebanon (37%) and Qatar (29%). General interest was similar in Lebanon (42%) and Qatar (45%), while Saudi Arabia showed slightly lower levels (42%). Preference for specific PGx topics differed across countries ($p<0.001$). Cancer chemotherapy emerged as a primary focus, particularly in Saudi Arabia (36%) and Lebanon (27%), with Qatar trailing at 15%. Lebanon showed the highest interest in complex diseases (28%). Cardiovascular diseases attracted significant attention in Qatar (20%) and Saudi Arabia (18%), while Lebanon showed lower interest (12%). Qatar uniquely prioritized diabetes mellitus (25%).

Participants were surveyed for their familiarity with the concept of PGx; in Qatar, 75% of the respondents were familiar with PGx, compared to 67% in Lebanon and 83% in Saudi Arabia (Figure 1A). In Qatar, 47% of respondents had not attended or given any talks on PGx, 8% had attended or given one, 31% had a few, and 14% had many (Figure 1B). Comparatively, 65% of respondents in Lebanon had not participated in any talks, 12% had participated in one, 16% in a few, and 7% in many (Figure 1B). In contrast, in Saudi Arabia, only 4% of respondents had not participated in any talks, 10% had participated in one, 40% in a few, and 36% in many (Figure 1B). Regarding PGx integration, 20% of Lebanon's respondents were not interested in integrating it into clinical practice, 66% considered it, 8% occasionally, and 2% routinely integrated PGx into their practice or research (Figure 1C). Similar trends were observed in Qatar and Saudi Arabia (Figure 1C).

Figure 2 illustrates the overall challenges faced in PGx implementation. The most significant challenge identified was test cost and reimbursement, highlighted by 13% of respondents. This was followed by insufficient physician knowledge or awareness, indicated by 9% of respondents. The unavailability of test technology, infrastructure, workforce, or experts was also a notable concern, affecting 8% of respondents. About 7% of respondents recognized a lack of funding, investment, or support and insufficient public understanding or awareness. Another prominent issue was unclear or lack of guidelines, cited by 6%. Four percent of respondents found that data from different populations or ethnicities, lack of adequate counselors, adequate communication of results, and limited evidence for clinical utility were significant barriers to PGx implementation in their countries.

The PGx challenges revealed significant hurdles, with varying levels of concern across the countries. Regarding test cost and reimbursement, Lebanon reported the highest concern, with 16% of respondents identifying it as a challenge, compared to 10% in Qatar and 5% in Saudi Arabia. Regarding the unavailability of test technology, infrastructure, workforce, or experts, 9% of respondents in Lebanon saw it as a challenge, higher than 6% in Qatar and 4% in Saudi Arabia. Participants from Lebanon and Qatar showed equal concern about insufficient physician knowledge or awareness, with 9% each, notably higher than the 4% in Saudi Arabia. The lack of funding, investment, or support was most pronounced in Lebanon at 9%, compared to 5% in Qatar and 3% in Saudi Arabia. Insufficient public understanding or awareness was identified as a hurdle primarily in Qatar by 8% of participants, followed closely by Lebanon at 7%. In comparison, only 1% of respondents in Saudi Arabia identified it as a challenge.

A comparative analysis of future trends and interest in PGx based on the job positions of participants is shown in Table 3. When asked if they believe pharmacogenomics has the potential to improve patient outcomes, 91% of academics, 69% of clinicians, and 88% of healthcare professionals responded affirmatively (χ2= 19, P=0.001). In contrast, a small fraction, 1% of academics and 4% of healthcare professionals reacted negatively, with none of the clinicians disagreeing. Additionally, 8% of academics, 31% of clinicians, and 8% of healthcare professionals were undecided. Interest in learning PGx varied across the groups, with 37% of academics, 59% of clinicians, and 44% of healthcare professionals expressing interest, and 49% of academics, 28% of clinicians, and 32% of healthcare professionals being very interested (χ2=15, P=0.02, Table 3). Only 1% of scholars and 4% of healthcare professionals were uninterested, while no clinicians expressed a lack of interest (χ2=15, P=0.02). Additionally, 13% of academics, 14% of clinicians, and 20% of healthcare professionals were slightly interested (χ2=15, P=0.02). The interest analysis according to the different age groups did not show any significant association (Supplementary Table). Regarding the PGx topics of interest, the responses varied but were not significantly different. The three job positions were mainly interested in complex diseases, cancer chemotherapy, cardiovascular diseases, and diabetes mellitus (χ2=31, P=0.06, Table 3).

Ninety percent of the PhD holders affirmed their belief in PGx and its potential to improve clinical outcomes (P<0.01, Table 4). The MD participants exhibited 70% affirmative and 30% undecided responses (P<0.01). The pharmacists had the highest affirmative rate at 97%, with 3% undecided and no negative responses (P<0.01). In contrast, Bachelor-level Healthcare professionals showed 85% affirmative responses, 10% undecided, and 5% negative (P<0.01). Among PhD holders, 50% expressed a very high level of interest in learning more about pharmacogenomics (PGx), 36% reported being interested, 13% were slightly interested, and 1% indicated no interest (P<0.01). MD respondents demonstrated 60% interest, with 27% being very interested, 13% slightly interested, and no disinterest reported (P<0.01). Pharmacists showed equal proportions of being very interested and interested (42% each), with 16% slightly interested and no reports of disinterest (P<0.01). Bachelor-level healthcare professionals displayed 44% interest, 28% very interested, 22% slightly interested, and 6% not interested (P<0.01). In terms of interest in specific PGx topics, PhD respondents reported the highest levels of interest in cancer chemotherapy (27%), complex diseases (18%), and infectious diseases (13%) (χ^2=41, P=0.06). MD participants showed considerable interest in cardiovascular diseases (21%) and cancer chemotherapy (20%), while pharmacists exhibited the highest interest in these two areas (25% and 21% respectively)

(Table 4). Bachelor-level healthcare professionals showed notable interest in cancer chemotherapy (21%) and complex diseases (19%), but interest in other topics remained lower across all groups (Table 4).

Discussion

While the impact of PGx on healthcare is substantial, the realities and expectations of precision medicine vary widely across different contexts and face numerous challenges. This study is the first to investigate the current status of PGx from a specialist viewpoint on a multinational level and is the first in the Arab world to provide a comparative perspective. The results suggest higher familiarity and educational involvement with PGx in Qatar and Saudi Arabia compared to Lebanon. However, a larger proportion of respondents in Lebanon are considering integrating PGx into their practice or research activities, while more respondents in Qatar and Saudi Arabia are already doing so occasionally or routinely.

Significant differences were noted in the level of uncertainty about PGx's potential and specific topics of interest, particularly regarding diabetes mellitus (Qatar) and cancer chemotherapy (Saudi Arabia). These differences are critical for tailoring educational and implementation strategies in each country, underscoring the variability in perceptions and needs for PGx across healthcare systems with different structures. To effectively implement precision medicine in clinical practice, it is crucial to evaluate the specific context of each healthcare system, including the current state of implementation, existing obstacles, and unique challenges.

PGx implementation in research and clinical practice in Arab and Middle Eastern countries faces several documented challenges. One significant challenge is the lag in biomedical research output in these countries (45). Another is the low genomic literacy rates among healthcare professionals, leading to a gap in translating genomic knowledge to patient care (46). This disparity can hinder the development and application of PGx knowledge, necessitating concerted efforts to bridge this gap (12). PGx implementation in Lebanon mirrors challenges faced by other nearby countries. Ongoing efforts to raise awareness among healthcare professionals in Lebanon (47) indicate growing interest in this field. However, limited availability, high costs of genetic testing, and delays in translating results underscore the need for solutions to these challenges (47).

PGx challenges varied between countries, highlighting the need for tailored strategies to address specific issues. Developing comprehensive training programs for healthcare professionals, including physicians and pharmacists, can enhance their understanding and utilization of PGx in clinical decision-making (48).

Addressing economic factors in Lebanon would require policy changes to improve accessibility and affordability of PGx testing, potentially through government subsidies or insurance coverage (46, 49, 50). In Qatar, where more respondents are already integrating PGx into their practice, developing robust clinical decision support systems and consultation services could facilitate regular use of PGx in patient care.

Several recommendations can be offered based on the study's findings. Firstly, educational and implementation strategies should be tailored to each country's needs and barriers (51). In Qatar, efforts should focus on transitioning from occasional or routine PGx integration to a more comprehensive and standardized approach. In Lebanon, targeted initiatives should support and incentivize PGx integration into practice or research activities. Addressing economic factors and raising awareness about PGx's potential benefits could encourage adoption in Lebanon and other countries.

In terms of PGx interest areas, specific attention should be given to diabetes mellitus in Qatar and cancer chemotherapy in Saudi Arabia, where there is significant interest due to the high incidence of certain cancers (52-55). Breast cancer is the most commonly diagnosed cancer among Lebanese and Saudi females, with a significant increase in incidence rates over the years (54, 56). Such high incidence of breast cancer in the two countries may justify the high interest in cancer PGx across participants from Saudi Arabia (36%) and from Lebanon (27%). Our findings are in parallel with global data on PGx of cancer therapies, where the oncology field has been leading on the forefront of precision medicine (57), and almost half of the FDA approvals for pharmacogenomic testing belong to the field of cancer therapeutics (58). When examining respondents' interest in PGx-specific topics, it becomes evident that certain diseases, such as complex diseases, cardiovascular diseases, and diabetes mellitus, may share overlapping etiology. Nevertheless, their pharmacogenomic implications and therapeutic strategies remain distinct. Internal and family medicine practitioners are often responsible for comprehensively managing complex diseases. In contrast, endocrinologists primarily address the intricate metabolic and hormonal aspects of diabetes mellitus, and cardiologists focus on the hemodynamic and structural complexities of cardiovascular diseases. By distinguishing these categories, we can capture clinicians' diverse and specialty-specific interests in PGx topics.

Collaboration with stakeholders, including healthcare professionals, educators, policymakers, and patients, is essential for successful PGx implementation (59). Engaging primary care physicians and pharmacists can enhance their knowledge and experience with PGx testing and facilitate its integration

into routine clinical practice (60). The importance of preemptive genotyping for precision medicine should be emphasized, as it can optimize medication selection and dosing, improving healthcare outcomes (61). Real-life examples of successful pharmacogenomic implementation include preemptive genotyping at institutions such as Vanderbilt and St. Jude Children's Research Hospital, which provides a basis for overcoming implementation challenges (62).

The stratified analysis according to the academic degree, showed interesting patterns (Table 4). Almost all PhD holders, pharmacists, and most MDs and bachelor-level healthcare professionals (>70%) believed that PGx could improve clinical outcomes. Interest in learning PGx varied significantly, with 50% of the PhDs being very interested, 60% of the MDs interested, and 42% and 28% of the pharmacists and bachelors (respectively) being very interested. Topic preferences were cancer chemotherapy (highest across groups), complex diseases (notable for PhDs and Bachelors), and cardiovascular diseases (preferred by MDs and Pharmacists).

Many clinicians can also have teaching activities; thus, their specialty areas might overlap with those of academics. We have carefully checked if the enrolled clinicians were also academics. Interestingly, almost all of them, except two from Lebanon, had teaching activities, and they specialize in PGx/Personalized Medicine and Medical Genetics. Interestingly, our academics had different specialties, hindering the need to analyze the data according to specialty area.

While the current results provide valuable insights into the differences between the three surveyed countries regarding familiarity, educational involvement, and challenges in integrating PGx into their healthcare systems, it is essential to highlight the limitations. First, the study's findings are based on self-reported data that can be subject to recall bias and social desirability bias, potentially influencing the accuracy of the responses. Second, the sample size and representativeness of respondents in the three countries should be considered. Third, the study may not have fully captured healthcare professionals' diverse perspectives and experiences in different practice settings, including those in rural or smaller healthcare facilities. Fourth, the survey did not gauge data on participant’s awareness of existing pharmacogenomic guidelines; such data would have provided valuable insights into their familiarity with standardized practices.

This study underscores the crucial need for education and training in PGx, as the lack of awareness and expertise among healthcare professionals can pose a significant barrier to integrating PGx information into clinical decision-making processes. Arab and Middle Eastern nations face multifaceted obstacles in advancing PGx research and integrating it into clinical practice. These challenges encompass resource constraints, educational gaps, regulatory hurdles, ethical considerations, data limitations, and economic factors. A comprehensive understanding of these challenges is crucial for devising effective strategies to overcome them.

References:

1. Lander ES, Linton LM, Birren B, Nusbaum C, Zody MC, Baldwin J, et al. Initial sequencing and analysis of the human genome. Nature. 2001;409(6822):860-921.
2. Dickmann LJ, Ware JA. Pharmacogenomics in the age of personalized medicine. Drug Discov Today Technol. 2016;21-22:11-6.
3. Singh DB. The Impact of Pharmacogenomics in Personalized Medicine. Adv Biochem Eng Biotechnol. 2020;171:369-94.
4. What is the difference between precision medicine and personalized medicine? What about pharmacogenomics? [Available from: https://medlineplus.gov/genetics/understanding/precisionmedicine/precisionvspersonalized/.
5. Delpierre C, Lefèvre T. Precision and personalized medicine: What their current definition says and silences about the model of health they promote. Implication for the development of personalized health. Front Sociol. 2023;8:1112159.
6. Sadee W, Wang D, Hartmann K, Toland AE. Pharmacogenomics: Driving Personalized Medicine. Pharmacol Rev. 2023;75(4):789-814.
7. US Food and Drug Administration. Table of valid genomic biomarkers in the context of approved drug labels 2024 [Available from: https://www.fda.gov/drugs/science-and-research-drugs/table-pharmacogenomic-biomarkers-drug-labeling.
8. Pirmohamed M. Pharmacogenomics: current status and future perspectives. Nat Rev Genet. 2023;24(6):350-62.
9. van der Lee M, Kriek M, Guchelaar HJ, Swen JJ. Technologies for Pharmacogenomics: A Review. Genes (Basel). 2020;11(12).
10. Sukri A, Salleh MZ, Masimirembwa C, Teh LK. A systematic review on the cost effectiveness of pharmacogenomics in developing countries: implementation challenges. Pharmacogenomics J. 2022;22(3):147-59.
11. Klein ME, Parvez MM, Shin JG. Clinical Implementation of Pharmacogenomics for Personalized Precision Medicine: Barriers and Solutions. J Pharm Sci. 2017;106(9):2368-79.
12. El Shamieh S, Zgheib NK. Pharmacogenetics in developing countries and low resource environments. Hum Genet. 2022;141(6):1159-64.
13. Zgheib NK, Patrinos GP, Akika R, Mahfouz R. Precision Medicine in Low- and Middle-Income Countries. Clin Pharmacol Ther. 2020;107(1):29-32.
14. Ayati N, Afzali M, Hasanzad M, Kebriaeezadeh A, Rajabzadeh A, Nikfar S. Pharmacogenomics Implementation and Hurdles to Overcome; In the Context of a Developing Country. Iran J Pharm Res. 2021;20(4):92-106.
15. Gameiro GR, Sinkunas V, Liguori GR, Auler-Júnior JOC. Precision Medicine: Changing the way we think about healthcare. Clinics (Sao Paulo). 2018;73:e723.
16. Zgheib NK. The Pharmacogenetics Laboratory of the Department of Pharmacology and Toxicology at the American University of Beirut Faculty of Medicine. Pharmacogenomics. 2017;18(14):1311-6.
17. Awada Z, Haider S, Tfayli A, Bazarbachi A, El-Saghir NS, Salem Z, et al. Pharmacogenomics variation in drug metabolizing enzymes and transporters in relation to docetaxel toxicity in Lebanese breast cancer patients: paving the way for OMICs in low and middle income countries. Omics. 2013;17(7):353-67.
18. Maamari D, El-Khoury H, Saifi O, Muwakkit SA, Zgheib NK. Implementation of Pharmacogenetics to Individualize Treatment Regimens for Children with Acute Lymphoblastic Leukemia. Pharmgenomics Pers Med. 2020;13:295-317.

19. Zgheib NK, Branch RA. Drug metabolism and liver disease: a drug-gene-environment interaction. Drug Metab Rev. 2017;49(1):35-55.
20. Zgheib NK, Alameddine R, Massoud R, Nasr R, Zahreddine A, El Cheikh J, et al. The role of candidate genetic polymorphisms in the interaction between voriconazole and cyclosporine in patients undergoing allogeneic hematopoietic cell transplantation: An explorative study. Curr Res Transl Med. 2020;68(2):51-8.
21. Saad H, Jabotian K, Sakr C, Mahfouz R, Akl IB, Zgheib NK. The Role of Angiotensin Converting Enzyme 1 Insertion/Deletion Genetic Polymorphism in the Risk and Severity of COVID-19 Infection. Front Med (Lausanne). 2021;8:798571.
22. Naja K, El Shamieh S, Fakhoury R. rs622342A>C in SLC22A1 is associated with metformin pharmacokinetics and glycemic response. Drug Metab Pharmacokinet. 2020;35(1):160-4.
23. Naja K, Salami A, El Shamieh S, Fakhoury R. rs622342 in SLC22A1, CYP2C9*2 and CYP2C9*3 and Glycemic Response in Individuals with Type 2 Diabetes Mellitus Receiving Metformin/Sulfonylurea Combination Therapy: 6-Month Follow-Up Study. J Pers Med. 2020;10(2).
24. Zgheib N, Ghaddar F, Sabra R. Teaching Pharmacogenetics on Low and Middle-Income Countries: Team Based Learning and Lessons Learned at The American University Of Beirut. Current Pharmacogenomics and Personalized Medicine. 2011;9:25-40.
25. Digihealth project: Elevating and Enriching a Pharmacogenomics course Through the Use of Open Education Resources 2021 [Available from: https://digihealth.uni-med.net/elevating-and-enriching-a-pharmacogenomics-course-through-the-use-of-open-education-resources/".
26. Zgheib NK, Arawi T, Mahfouz RA, Sabra R. Attitudes of health care professionals toward pharmacogenetic testing. Mol Diagn Ther. 2011;15(2):115-22.
27. Mehtar M, Hammoud SH, Amin MEK. An objective evaluation of fundamental pharmacogenomics knowledge among pharmacists and pharmacy students. Saudi Pharm J. 2022;30(12):1765-72.
28. Abou Diwan E, Zeitoun RI, Abou Haidar L, Cascorbi I, Khoueiry Zgheib N. Implementation and obstacles of pharmacogenetics in clinical practice: An international survey. Br J Clin Pharmacol. 2019;85(9):2076-88.
29. Bastaki K, Velayutham D, Irfan A, Adnan M, Mohammed S, Mbarek H, et al. Forging the path to precision medicine in Qatar: a public health perspective on pharmacogenomics initiatives. Front Public Health. 2024;12:1364221.
30. Hendi NN, Al-Sarraj Y, Ismail Umlai UK, Suhre K, Nemer G, Albagha O. Genetic determinants of Vitamin D deficiency in the Middle Eastern Qatari population: a genome-wide association study. Front Nutr. 2023;10:1242257.
31. Velayutham D, Bastaki K, Ghuloum S, Azeem MW, Pirmohamed M, Jithesh PV. Clinically actionable pharmacogenomic landscape of antidepressants and antipsychotics in Qatar: A population-based cohort study. medRxiv. 2023:2023.09.27.23296201.
32. Bastaki K, El Anbari M, Ghuloum S, Jithesh PV. Prescription Pattern of Antidepressants and the Potential for Personalized Medicine in the Qatari Population. J Pers Med. 2021;11(5).
33. Jithesh PV, Abuhaliqa M, Syed N, Ahmed I, El Anbari M, Bastaki K, et al. A population study of clinically actionable genetic variation affecting drug response from the Middle East. NPJ Genom Med. 2022;7(1):10.
34. Ahmed I, Ziab M, Mbarak H, Taheri S, Chagoury O, Hussain SA, et al. Improved Type 2 Diabetes Risk Stratification in the Qatar Biobank Cohort by Ensemble Learning Classifier Incorporating Multi-Trait, Population-Specific, Polygenic Risk Scores. medRxiv. 2023:2023.06.23.23291830.
35. Qatar Genome publications [Available from: https://www.qatargenome.org.qa/research/publications.

36. Elewa H, Alkhiyami D, Alsahan D, Abdel-Aziz A. A survey on the awareness and attitude of pharmacists and doctors towards the application of pharmacogenomics and its challenges in Qatar. J Eval Clin Pract. 2015;21(4):703-9.
37. Alrabiah Z, Syed W, Babelghaith SD, Al Arifi MN. Clinical Knowledge, Attitude, and Perceptions of Community Pharmacists Towards Pharmacogenomics - A Cross-Sectional Study from Saudi Arabia. Pharmgenomics Pers Med. 2023;16:433-41.
38. Mensah GA, Fuster V, Murray CJL, Roth GA. Global Burden of Cardiovascular Diseases and Risks, 1990-2022. J Am Coll Cardiol. 2023;82(25):2350-473.
39. Goljan E, Abouelhoda M, Tahir A, ElKalioby M, Meyer B, Monies D. Large-scale next generation sequencing based analysis of SLCO1B1 pharmacogenetics variants in the Saudi population. Hum Genomics. 2024;18(1):30.
40. Alhazzani AA, Munisamy M, Karunakaran G. Pharmacogenetics of CYP2C19 genetic polymorphism on clopidogrel response in patients with ischemic stroke from Saudi Arabia. Neurosciences (Riyadh). 2017;22(1):31-7.
41. Swen JJ, Nijenhuis M, van Rhenen M, de Boer-Veger NJ, Buunk AM, Houwink EJF, et al. Pharmacogenetic Information in Clinical Guidelines: The European Perspective. Clin Pharmacol Ther. 2018;103(5):795-801.
42. Algahtani M. Knowledge, Perception, and Application of Pharmacogenomics Among Hospital Pharmacists in Saudi Arabia. Risk Manag Healthc Policy. 2020;13:1279-91.
43. Cuschieri S. The STROBE guidelines. Saudi J Anaesth. 2019;13(Suppl 1):S31-s4.
44. Ghaddar F, Cascorbi I, Zgheib NK. Clinical implementation of pharmacogenetics: a nonrepresentative explorative survey to participants of WorldPharma 2010. Pharmacogenomics. 2011;12(7):1051-9.
45. Benamer HT, Bakoush O. Arab nations lagging behind other Middle Eastern countries in biomedical research: a comparative study. BMC Med Res Methodol. 2009;9:26.
46. Hippman C, Nislow C. Pharmacogenomic Testing: Clinical Evidence and Implementation Challenges. J Pers Med. 2019;9(3).
47. Ossaily S, Zgheib NK. The pharmacogenetics of drug metabolizing enzymes in the Lebanese population. Drug Metabol Drug Interact. 2014;29(2):81-90.
48. Johansen Taber KA, Dickinson BD. Pharmacogenomic knowledge gaps and educational resource needs among physicians in selected specialties. Pharmgenomics Pers Med. 2014;7:145-62.
49. The World Bank In Lebanon [updated Nov 02, 2022. Available from: https://www.worldbank.org/en/country/lebanon/overview.
50. Bou Sanayeh E, El Chamieh C. The fragile healthcare system in Lebanon: sounding the alarm about its possible collapse. Health Econ Rev. 2023;13(1):21.
51. Roman YM. Editorial: The role of pharmacogenomics in addressing health disparities: the path, the promise, and the barriers. Front Genet. 2023;14:1233045.
52. Qattan MY. The Incidence Rate of Non-Hodgkin's Lymphoma in Saudi Arabia: An Observational Descriptive Population Based Epidemiological Study from the Saudi Cancer Registry (2006–2016). Open Access Macedonian Journal of Medical Sciences. 2023;11(B):445-52.
53. Alsalem A, Alessa M, Alshaalan Y, Alkahtani N, Alamoudi M. Trends in the Prevalence and Incidence of Thyroid Cancer in Saudi Arabia Over the Last Decade: A Retrospective Cohort Study of 9882 Patients. 2023.
54. Basudan AM. Breast Cancer Incidence Patterns in the Saudi Female Population: A 17-Year Retrospective Analysis. Medicina (Kaunas). 2022;58(11).
55. Alghamdi IG. Epidemiology of gastric cancer in Saudi Arabia from 2004 to 2017. Mol Clin Oncol. 2023;19(5):93.

56. Fares MY, Salhab HA, Khachfe HH, Khachfe HM. Breast Cancer Epidemiology among Lebanese Women: An 11-Year Analysis. Medicina (Kaunas). 2019;55(8).
57. Saadeh C, Bright D, Rustem D. Precision Medicine in Oncology Pharmacy Practice. Acta Med Acad. 2019;48(1):90-104.
58. Kim JA, Ceccarelli R, Lu CY. Pharmacogenomic Biomarkers in US FDA-Approved Drug Labels (2000-2020). J Pers Med. 2021;11(3).
59. Bank PCD, Swen JJ, Guchelaar HJ. Implementation of Pharmacogenomics in Everyday Clinical Settings. Adv Pharmacol. 2018;83:219-46.
60. Haga SB. The Critical Role of Pharmacists in the Clinical Delivery of Pharmacogenetics in the U.S. Pharmacy (Basel). 2023;11(5).
61. Gallaway KA, Cann K, Oetting K, Rothenberger M, Raibulet A, Slaven JE, et al. The Potential Impact of Preemptive Pharmacogenetic Genotyping in the Neonatal Intensive Care Unit. J Pediatr. 2023;259:113489.
62. Rafi I, Crinson I, Dawes M, Rafi D, Pirmohamed M, Walter FM. The implementation of pharmacogenomics into UK general practice: a qualitative study exploring barriers, challenges and opportunities. J Community Genet. 2020;11(3):269-77.

Table 1: Demographic characteristics of the study participants in Lebanon, Qatar, and Saudi Arabia.

**Demographic characteristics**	**All (N=337)**		**Lebanon (N=145)**		**Qatar (N=137)**		**Saudi Arabia (N=55)**	
**Age categories N(%)**								
18-25	103	(30%)	95	(66%)	6	(4.5%)	2	(4%)
26-35	84	(25%)	26	(18%)	45	(33%)	13	(24%)
36-45	105	(32%)	15	(10%)	58	(42%)	32	(57%)
46-55	35	(10%)	6	(4%)	22	(16%)	7	(13%)
> 55	10	(3%)	3	(2%)	6	(4.5%)	1	(2%)
**Gender N(%)**								
Male	118	(35%)	49	(34%)	41	(30%)	28	(51%)
Female	219	(65%)	96	(66%)	96	(70%)	27	(49%)
**Job N(%)**								
Academic	106	(32%)	33	(23%)	34	(25%)	39	(70%)
Clinician	29	(9%)	9	(6%)	13	(9%)	8	(15%)
Healthcare Professional	202	(59%)	103	(71%)	90	(66%)	8	(15%)
Academic Degree N(%)								
PhD	101	(30%)	29	(20%)	34	(25%)	38	(69%)
MD	30	(9%)	10	(7%)	13	(9%)	7	(13%)
BS in Pharmacy	64	(19%)	9	(6%)	45	(33%)	10	(18%)
Bachelor	142	(42%)	97	(67%)	45	(33%)	0	
**Professional Experience N(%)**								
< 1 year	62	(18%)	39	(27%)	17	(12%)	6	(11%)
1-5 years	115	(34%)	72	(50%)	34	(25%)	9	(16%)
6-10 years	57	(17%)	12	(8%)	27	(20%)	18	(33%)
11-20 years	74	(22%)	17	(12%)	42	(31%)	15	(27%)
> 20 years	29	(9%)	5	(3%)	17	(12%)	7	(13%)

For continuous variables, values are the arithmetic mean ± SD. Categorical variables are shown as numbers (n) and percentages (%).
n: sample size.

Table 2: Future directions and interest in pharmacogenomics in Lebanon, Qatar, and Saudi Arabia.

	Lebanon		Qatar		Saudi Arabia		χ2	P
**Do you believe that pharmacogenomics has the potential to significantly improve patient outcomes in clinical practice?**								
No	6	(4%)	3	(2%)	0			
Yes	125	(86%)	117	(86%)	52	(94%)	5	0.278
Undecided	14	(10%)	17	(12%)	3	(6%)		
**How interested would you be in learning about pharmacogenomics?**								
Not Interested	3	(2%)	7	(5%)	0			
Slightly Interested	27	(19%)	29	(21%)	2	(4%)		
Interested	61	(42%)	61	(45%)	23	(42%)	18	0.005
Very Interested	54	(37%)	40	(29%)	30	(55%)		
**What specific pharmacogenomics topics, if any, would you like to learn about?**								
Cancer chemotherapy	39	(27%)	21	(15%)	20	(36%)		
Cardiovascular diseases	18	(12%)	27	(20%)	10	(18%)		
Complex diseases*	41	(28%)	4	(3%)	12	(22%)		
Diabetes Mellitus	0		34	(25%)	0			
Immune Suppressants	0		9	(7%)	0			
Infectious diseases	15	(10%)	11	(8%)	4	(7%)6	141	<0.001
Neurodegenerative disorders	8	(6%)	9	(6%)	7	(13%)		
Pain management	0		12	(9%)	0			
Precision vaccines and immunity	8	(6%)	0		0			
Psychiatric disorders	12	(8%)	10	(7%)	0			
Rare diseases	4	(3%)	0	0	2	(4%)		

* Complex diseases are common diseases caused by a combination of genetic, environmental, and lifestyle factors.

For continuous variables, values are the arithmetic mean ± SD.

Categorical variables are shown as numbers (n) and percentages (%).n: sample size. A χ2 test of independence was used to test if the differences between Lebanon and water are significantly different.

Table 3: Future directions and Interest in pharmacogenomics according to participants' jobs.

	**Academic**		**Clinician**		**Healthcare professional**		**χ2**	**P**
**Do you believe that pharmacogenomics has the potential to significantly improve patient outcomes in clinical practice?**								
No	1	(1%)	0		8	(4%)		
Undecided	9	(8%)	9	(31%)	16	(8%)	19	0.001
Yes	96	(91%)	20	(69%)	178	(88%)		
**How interested would you be in learning about pharmacogenomics?**								
Interested	39	(37%)	17	(59%)	89	(44%)		
Not interested	1	(1%)	0	0	9	(4%)	15	0.02
Slightly	14	(13%)	4	(14%)	40	(20%)		
Very Interested	52	(49%)	8	(28%)	64	(32%)		
**What specific pharmacogenomics topics, if any, would you like to learn about?**								
All the complex diseases*	19	(18%)	5	(17%)	33	(16%)		
Cancer chemotherapy	29	(27%)	6	(21%)	45	(22%)		
Cardiovascular diseases	13	(12%)	6	(21%)	36	(18%)		
Diabetes Mellitus	5	(5%)	4	(14%)	25	(12%)		
Immune Suppressants	2	(2%)	1	(3%)	6	(3%)		
Infectious diseases	14	(13%)	3	(10%)	13	(6%)	31	0.06
Neurodegenerative disorders	12	(11%)	0		12	(6%)		
Pain management	2	(2%)	0		10	(5%)		
Personalized vaccines and immunity	4	(4%)	0		4	(2%)		
Psychiatric disorders	3	(3%)	2	(7%)	17	(8%)		
Rare diseases	3	(3%)	2	(7%)	1	(0%)		

* Complex diseases are common diseases caused by a combination of genetic, environmental, and lifestyle factors.

For continuous variables, values are the arithmetic mean ± SD. Categorical variables are shown as numbers (n) and percentages (%). n: sample size. A χ2 test of independence was used to test if the differences between Lebanon and water are significantly different.

Table 4: Future directions and Interest in pharmacogenomics according to the academic Degree.

	PhD		MD		Bachelor in Pharmacy		Bachelor		χ2	P
No	1	(1%)			0		8	(5%)		
Undecided	9	(9%)	9	(30%)	2	(3%)	14	(10%)	25	<0.01
Yes	91	(90%)	21	(70%)	62	(97%)	120	(85%)		
Interested	37	(36%)	18	(60%)	27	(42%)	63	(44%)		
Not interested	1	(1%)			0		9	(6%)		<0.01
Slightly	13	(13%)	4	(13%)	10	(16%)	31	(22%)	24	
Very Interested	50	(50%)	8	(27%)	27	(42%)	39	(28%)		
All the complex diseases*	18	(18%)	5	(17%)	5	(8%)	29	(19%)		
Cancer chemotherapy	28	(27%)	6	(20%)	15	(21%)	32	(21%)		
Cardiovascular diseases	13	(13%)	6	(21%)	16	(25%)	20	(14%)		
Diabetes Mellitus	5	(5%)	4	(13%)	4	(6%)	21	(14%)		
Immune Suppressants	2	(2%)	1	(3%)	3	(5%)	3	(2%)		
Infectious diseases	13	(13%)	3	(10%)	4	(6%)	10	(5%)		
Neurodegenerative disorders	11	(11%)	1	(3%)	6	(10%)	6	(3%)	4S1	0.06
Pain management	2	(2%)			5	(8%)	5	(2%)		
Personalized vaccines and immunity	3	(3%)			2	(3%)	3	(12%)		
Psychiatric disorders	3	(3%)	2	(7%)	5	(8%)	12	(7%)		
Rare diseases	3	(3%)	2	(7%)	0		1	(1%)		

* Complex diseases are common diseases caused by a combination of genetic, environmental, and lifestyle factors.

For continuous variables, values are the arithmetic mean ± SD. Categorical variables are shown as numbers (n) and percentages (%). n: sample size. A χ2 test of independence was used to test if the differences between Lebanon and water are significantly different.

Figure 1: Knowledge and attitudes towards implementation of pharmacogenomics in clinical practice and research.

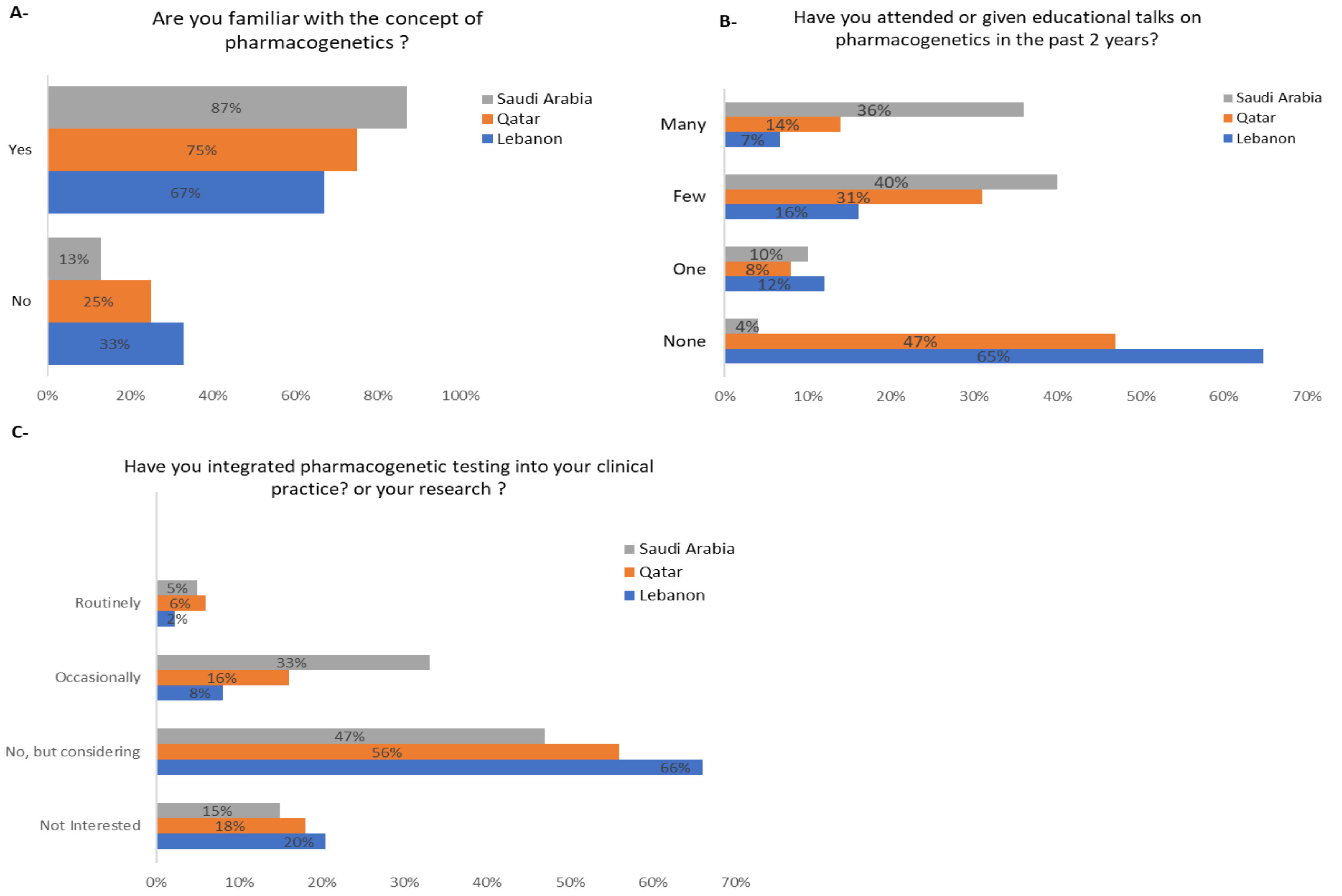

A: Familiarity with the Concept of Pharmacogenomics. B. Attendance or Presentation of Educational Talks on Pharmacogenomics in the Past 2 Years. C. Integration of Pharmacogenomics into Clinical Practice or Research Activities.

Figure 2: The overall Challenges for bringing pharmacogenetics into clinical practice.

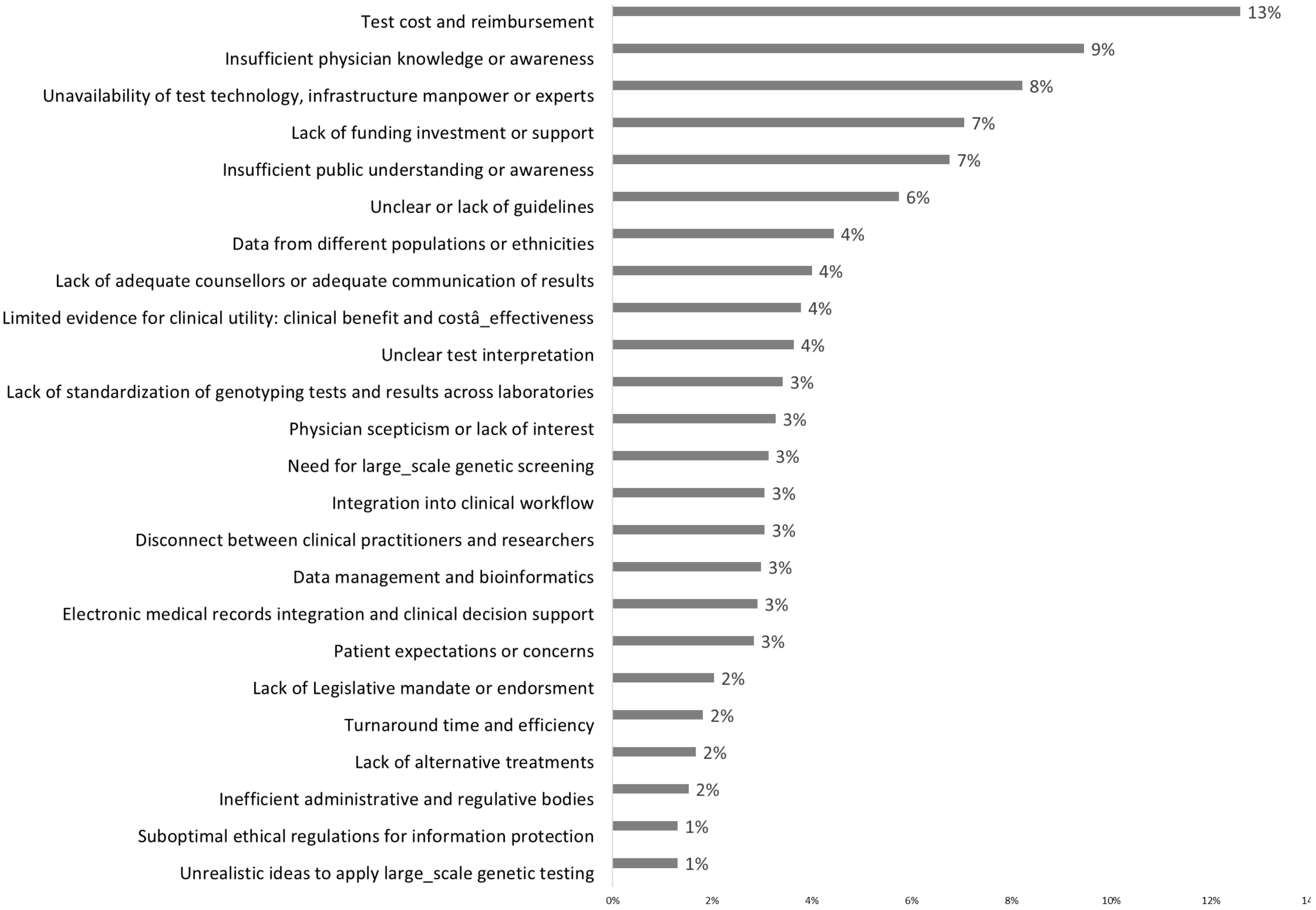

Figure 3: Challenges for bringing pharmacogenetics into clinical practice in Lebanon, Qatar, and Saudi Arabia.

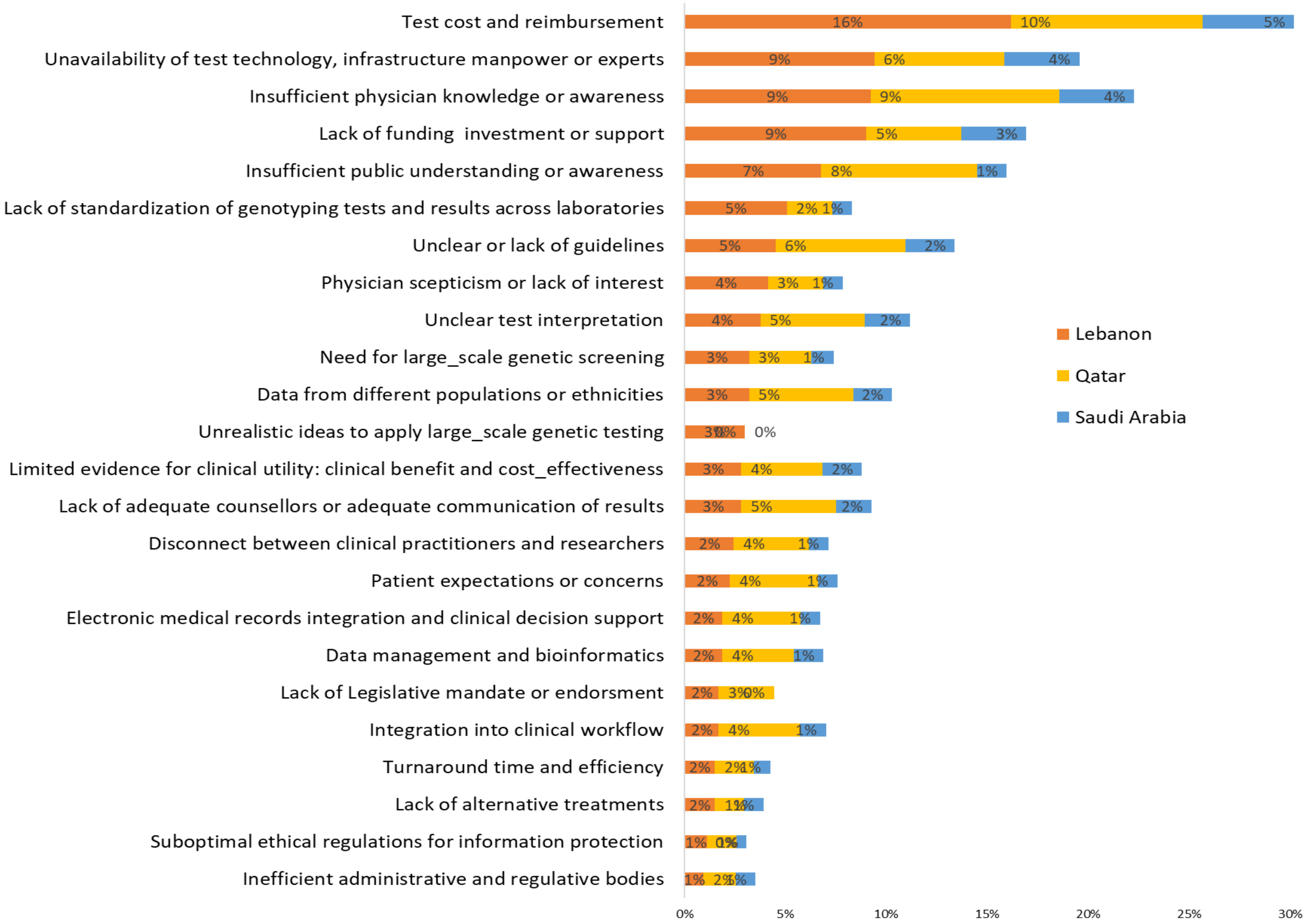

Click here to access/download
Supporting Information
Supplementary table.docx

Integrating Pharmacogenomics in Three Middle Eastern Countries' Healthcare (Lebanon, Qatar, and Saudi Arabia)~~Middle Eastern Healthcare~~: Current Insights, Challenges, and Strategic Directions

Said El Shamieh[1*], Rimah Abdullah Saleem[2*], Dalal Hammoudi Halat[3], Hana M A Fakhoury[2], Kholoud Bastaki[4], Mirna Fawaz[5], Ahmed Malki[3], Rajaa Fakhoury[1,2 #].

[*] Contributed equally

[1] Department of Medical Laboratory Technology, Faculty of Health Sciences, Beirut Arab University, Beirut 11-5020, Lebanon.

[2] Department of Biochemistry and Molecular Medicine, College of Medicine, Alfaisal University, Riyadh, Saudi Arabia.

[3] Academic Quality Department, QU Health, Qatar University, P.O. Box 2713, Doha, Qatar.

[4] Clinical and Pharmacy Practice Department, College of Pharmacy, QU Health, Qatar University, Doha, Qatar.

[5] Department of Nursing, Faculty of Health Sciences, Beirut Arab University, Beirut 11-5020, Lebanon.

[#] Corresponding author: Rajaa Fakhoury rfakhoury@bau.edu.lb

Abstract

Background and Objectives: Pharmacogenomics (PGx) leverages genomic information to tailor drug therapies, enhancing precision medicine. Despite global advancements, its implementation in Lebanon, Qatar, and Saudi Arabia faces unique challenges in clinical integration. This study aimed to investigate PGx attitudes, knowledge implementation, associated challenges, forecast future educational needs, and compare findings across the three countries.

Methods: This cross-sectional study utilized an anonymous, self-administered online survey distributed to healthcare professionals, academics, and clinicians in Lebanon, Qatar, and Saudi Arabia. The survey comprised 18 questions ~~designed~~ to assess participants' familiarity with PGx, current implementation practices, perceived obstacles, potential integration strategies, and future educational needs.

Results: The survey yielded 337 responses from healthcare professionals across the three countries. Data revealed significant variations in PGx familiarity and educational involvement. Qatar and Saudi Arabia participants were more ~~showed higher~~ familiar~~ity~~ with PGx compared to Lebanon (83%, 75%, and 67%, respectively). Participation in PGx-related talks was most prevalent in Saudi Arabia (96%), followed by Qatar (53%) and Lebanon (35%). Key challenges identified included test cost and reimbursement, insufficient physician knowledge, and lack of infrastructure. Lebanon reported the highest concern for test costs (16%), compared to the lowest in Saudi Arabia (5%). Despite these challenges, a strong consensus emerged on PGx's potential to improve patient outcomes, with over 86% of respondents in all three countries expressing this belief. Educational interest areas varied by country, with strong interest in PGx for cancer chemotherapy in Saudi Arabia and Lebanon and for diabetes mellitus in Qatar.

Conclusion: This study highlights the significant influence of varied educational backgrounds and infrastructural limitations on PGx implementation across Lebanon, Qatar, and Saudi Arabia. The findings emphasize the need for targeted strategies in each country to address these distinct barriers. Integrating PGx education into healthcare training programs and clinical workflows could unlock PGx's potential to optimize patient care.

Keywords: Pharmacogenomics, Lebanon, Qatar, Saudi Arabia, precision medicine.

Introduction

The completion of the human genome sequencing project in 2001 marked a pivotal moment in biomedical research, laying the foundation for the field of pharmacogenomics (PGx) (1). PGx examines the influence of an individual's genetic makeup on their response to drugs, using DNA signatures to guide treatment and inform drug development and testing (2). Recent advances in PGx have deepened our understanding of the factors contributing to individual variations in drug response. Genetic variants correlated with drug responses can now be utilized to tailor drug selection and dosing for each patient (3). As a cornerstone of precision medicine, PGx integrates genomic, environmental, and lifestyle information to inform medical management decisions, providing a more precise approach to disease prevention, diagnosis, and treatment (4-6). The increasing number of US FDA approvals for precision therapeutics underscores the growing impact of PGx (7). However, several challenges impede its routine implementation, including modifications of current clinical practices, knowledge gaps, a lack of interest among healthcare professionals, technological hurdles, and cost-effectiveness issues, particularly in developing countries (8-11).

The literature highlights a disparity in PGx research and implementation between low-resource settings and developed economies, each facing unique obstacles and needs (12, 13). There is a significant need for education and training among healthcare providers to effectively interpret and apply PGx data in clinical settings. This includes integrating PGx into medical curricula and continuous professional development programs (14, 15). In Lebanon, efforts in PGx have been progressing, with several pharmacogenetics laboratories established in the past decade (16). Studies employing next-generation sequencing and other genotyping techniques demonstrate active participation in this field (17-23). However, an international survey of PGx experts, ~~which included Lebanon, indicated that despite the availability of PGx tests and a positive attitude towards their applications, clinical implementation remains challenging~~including Lebanon, indicated that clinical implementation remains challenging despite the availability of PGx tests and a positive attitude towards their applications. Moreover, while PGx education is included in the curricula of Lebanese higher education institutions (24, 25), there is still a need for further education to improve foundational knowledge and capitalize on significant opportunities for advancement (26-28).

In contrast, Qatar has made considerable strides in PGx, supported by an advanced healthcare infrastructure, a national genome program, and numerous educational initiatives focused on precision medicine (29). Research in Qatar encompasses a variety of pharmacogenomic applications, including

antipsychotics, antidepressants, immunosuppressants, warfarin, vitamin D deficiency, and type 2 diabetes risk stratification. To support PGx implementation, Qatar has undertaken numerous educational initiatives, including courses for healthcare providers, conferences, curricular updates, and the establishment of precision medicine committees at institutional and national levels (29-35). To support PGx implementation, Qatar has undertaken numerous educational initiatives, including courses for healthcare providers, conferences, curricular updates, and the establishment of precision medicine committees at institutional and national levels (29). Studies indicate that physicians and pharmacists in Qatar possess positive attitudes and are motivated to engage in PGx-related clinical applications and patient education (36).

PGx is an emerging field in Saudi Arabia with significant implications for personalized medicine. However, there is a notable lack of knowledge and understanding of PGx among healthcare professionals, including community pharmacists and physicians. (37). Research shows that many individuals in the local population carry PGx variants, which affect a substantial percentage of medications dispensed annually, highlighting the need for tailored prescription practices (38-40).

Despite the rapid growth of PGx literature and increasing support from regulatory agencies, there remains a gap in the literature regarding its actual status and implementation (41). Enhancing education and training initiatives to include pharmacogenomics, improving healthcare professionals' expertise, and raising awareness among policymakers about the importance of PGx could leverage future insights for better healthcare applications (42). Focused exploration of PGx attitudes, knowledge, implementation, and associated barriers and needs in Lebanon, Qatar, and Saudi Arabia would be instrumental in unlocking PGx's full potential in improving patient care and healthcare systems. To our knowledge, a consolidated investigation of these PGx pillars from the perspective of healthcare professionals and specialists has not been previously undertaken. This study aims to examine the status of PGx implementation in Lebanon, Qatar, and Saudi Arabia, identify associated challenges, predict future educational needs, and compare findings across these three countries.

Methodology

Ethical statement and informed consent

Ethical approval for this study was obtained from the institutional review boards of the participating universities: Beirut Arab University (2023-H-0153-HS-R-0545), Qatar University (QU-IRB 1995-E/23), and Alfaisal University (IRB-20270). Informed consent was obtained from all participants online, ensuring their confidentiality and the right to withdraw from the study without any consequences. Participants were informed that all collected data would be anonymous and confidential, with only the principal investigator having access to the data. Completing and submitting the survey was considered an agreement to participate.

Study design

This study utilized a quantitative cross-sectional research design, involving healthcare professionals (pharmacists, nurses, medical laboratory technologists), university academics, and clinicians from Lebanon, Qatar, and Saudi Arabia. Data was collected through a voluntary, anonymous, private survey to gather PGx perspectives from specialists in the three countries. The study was conducted and reported following the Strengthening the Reporting of Observational Studies in Epidemiology (STROBE) guidelines (43).

Survey instrument

The survey questions were adapted from the previously published manuscript by of Ghaddar et al. , with the approval of Pr. Nathalie Zgheib (44). The instrument comprised 18 questions divided into several sections. The first section collected demographic information such as age, gender, country of residence, occupation, and years of experience in clinical practice or research. The second section focused on PGx implementation, examining participants' familiarity with the concept, attendance or presentation of educational talks, and integration of pharmacogenetic testing into their practice or research. The third section explored the obstacles faced in implementing PGx, asking participants to identify the top five obstacles from a provided list. The final section inquired about future considerations, including measures to overcome barriers in PGx implementation, belief in the potential of PGx to improve patient outcomes, interest in learning about pharmacogenomics, and specific topics of interest.

Before launching, the survey was pilot-tested with a small group of healthcare professionals to ensure content clarity and relevance. Based on the pilot test, the survey questions were modified, added, or deleted to improve the content. The pilot responses were not included in the final analysis. The survey items were tailored to country-specific practice norms through consultation with experts from Lebanon and Qatar, where data collection began. For instance, the PGx of pain management was added due to its common testing in Qatari patients.

Data collection

Participants were recruited through purposive sampling, targeting professionals directly or indirectly involved in PGx. PGx opinion leaders or experts in each country were approached personally via direct email or social media and asked to complete and disseminate the survey within their teams. The survey was launched on September 27th, 2023, and data collection ended on December 23rd, 2023, in Lebanon; January 24th, 2024, in Qatar; and June 7th, 2024, in Saudi Arabia. Participants were briefed about the study's aim in the survey's introductory paragraph and directed to respond anonymously through Google Forms. The survey took approximately 4 minutes to complete.

Statistical analysis

All statistical analyses were performed using SPSS software (version 26; SPSS Inc., Chicago, IL). Descriptive statistics were used to summarize demographic characteristics and survey responses, with categorical variables expressed as frequencies and percentages. Group comparisons were conducted using the chi-square (χ^2) test of independence to assess whether there were significant differences across the study groups; (1) countries, (2) participants' jobs, (3) academic degrees. The measurable variables included demographic characteristics such as age categories, gender, professional roles, and years of professional experience. Familiarity and involvement with PGx were also compared across the study groups, including familiarity with the concept of PGx, attendance or presentation of educational talks on PGx in the past two years, and the integration of PGx into clinical or research practices. Additionally, future interest and educational needs were assessed by comparing beliefs in PGx's potential to improve patient outcomes, levels of interest in learning about PGx, and specific PGx topics of interest. For each measurable variable, contingency tables were created to cross-tabulate responses by study group, and the χ^2 test was applied to assess independence between these variables. The statistical significance threshold was set at $P \leq 0.05$. When expected cell counts in the contingency table were less than 5, Fisher's exact test was used instead

to ensure the validity of the results. Results are reported with the corresponding χ², and P-values to provide transparency and context for the observed differences. All analyses were conducted using SPSS software version 26 (SPSS, Inc., Chicago, Illinois). Variables were treated as categorical and expressed as percentages. The chi-square (χ2) test of independence was used to test differences in proportions between categorical variables, with the significance level set at P≤0.05.

Results

The demographic characteristics of the study participants from Lebanon, Qatar, and Saudi Arabia are detailed in Table 1. The total sample comprised 337 participants: 145 (43%) from Lebanon, 137 (41%) from Qatar, and 55 (16%) from Saudi Arabia. Age distribution showed a significant skew, with a larger proportion of younger participants (18-25 years) in Lebanon (66%) compared to Qatar (4.5%) and Saudi Arabia (4%). Middle-aged groups (26-45 years) were more prevalent in Qatar, while 57% of respondents from Saudi Arabia were between 36 and 45 years old. Gender distribution was similar across the countries, with females constituting the majority: 66% in Lebanon, 70% in Qatar, and 51% in Saudi Arabia. Academics and clinicians were similarly distributed between Qatar and Lebanon. However, there was a notable disparity in healthcare professionals, with Lebanon having a slightly higher proportion (71% vs. 66% in Qatar, Table 1). In terms of academic degrees, the PhD holders represented 30% of our participants, primarily in Saudi Arabia (69%), with Qatar (25%) and Lebanon (20%) trailing. MD holders were 9%, with distributions of 13% in Saudi Arabia, 9% in Qatar, and 7% in Lebanon. BS in Pharmacy holders made up 19%, dominated by Qatar (33%), followed by Saudi Arabia (18%) and Lebanon (6%). The Bachelor's degree (healthcare professionals), the largest group at 42%, were concentrated in Lebanon (67%) and Qatar (33%), with none in Saudi Arabia. Professional experience varied, with Qatar and Saudi Arabia showing higher representation in the more experienced categories than Lebanon (11-20 years and more than 20 years).

The perspectives on PGx across the three countries regarding beliefs, educational interests, and preferred topics were also analyzed (Table 2). Participants from all three countries agreed on the potential of PGx to improve patient outcomes, with no significant differences across the three study groups. Interest in learning about PGx varied significantly among the regions (P<0.005, Table 2). Saudi Arabia recorded the highest proportion of participants very interested in PGx (55%), compared to Lebanon (37%) and Qatar (29%). General interest was similar in Lebanon (42%) and Qatar (45%), while Saudi Arabia showed slightly lower levels (42%). Preference for specific PGx topics differed across countries (p<0.001). Cancer chemotherapy emerged as a primary focus, particularly in Saudi Arabia (36%) and Lebanon (27%), with Qatar trailing at 15%. Lebanon showed the highest interest in complex diseases (28%). Cardiovascular diseases attracted significant attention in Qatar (20%) and Saudi Arabia (18%), while Lebanon showed lower interest (12%). Qatar uniquely prioritized diabetes mellitus (25%).

Participants were surveyed for their familiarity with the concept of PGx; in Qatar, 75% of the respondents were familiar with PGx, compared to 67% in Lebanon and 83% in Saudi Arabia (Figure 1A). In Qatar, 47% of respondents had not attended or given any talks on PGx, 8% had attended or given one, 31% ~~a few, and 14%~~ had a few, and 14% had many (Figure 1B). Comparatively, 65% of respondents in Lebanon had not participated in any talks, 12% had participated in one, 16% in a few, and 7% in many (Figure 1B). In contrast, in Saudi Arabia, only 4% of respondents had not participated in any talks, 10% had participated in one, 40% in a few, and 36% in many (Figure 1B). Regarding PGx integration, 20% of Lebanon's respondents were not interested in integrating it into clinical practice, 66% ~~were considering~~considered it, 8% occasionally, and 2% routinely integrated PGx into their practice or research (Figure 1C). Similar trends were observed in Qatar and Saudi Arabia (Figure 1C).

Figure 2 illustrates the overall challenges faced in PGx implementation. The most significant challenge identified was test cost and reimbursement, highlighted by 13% of respondents. This was followed by insufficient physician knowledge or awareness, indicated by 9% of respondents. The unavailability of test technology, infrastructure, workforce, or experts was also a notable concern, affecting 8% of respondents. About 7% of respondents recognized a lack of funding, investment, or support and insufficient public understanding or awareness. Another prominent issue was unclear or lack of guidelines, cited by 6%. Four percent of respondents found that data from different populations or ethnicities, lack of adequate counselors, adequate communication of results, and limited evidence for clinical utility were significant barriers to PGx implementation in their countries.

The PGx challenges revealed significant hurdles, with varying levels of concern across the countries. Regarding test cost and reimbursement, Lebanon reported the highest concern, with 16% of respondents identifying it as a challenge, compared to 10% in Qatar and 5% in Saudi Arabia. Regarding the unavailability of test technology, infrastructure, workforce, or experts, 9% of respondents in Lebanon saw it as a challenge, higher than 6% in Qatar and 4% in Saudi Arabia. Participants from Lebanon and Qatar showed equal concern about insufficient physician knowledge or awareness, with 9% each, notably higher than the 4% in Saudi Arabia. The lack of funding, investment, or support was most pronounced in Lebanon at 9%, compared to 5% in Qatar and 3% in Saudi Arabia. Insufficient public understanding or awareness was identified as a hurdle primarily in Qatar by 8% of participants, followed closely by Lebanon at 7%. In comparison, only 1% of respondents in Saudi Arabia identified it as a challenge.

~~Table 3 presents a~~ A comparative analysis of future trends and interest in PGx based on the job positions of participants~~: academics, clinicians, and healthcare professionals~~ is shown in Table 3. When asked if they believe pharmacogenomics has the potential to improve patient outcomes, 91% of academics, 69% of clinicians, and 88% of healthcare professionals responded affirmatively (χ2= 19, P=0.001). In contrast, a small fraction, 1% of academics and 4% of healthcare professionals reacted negatively, with none of the clinicians disagreeing. Additionally, 8% of academics, 31% of clinicians, and 8% of healthcare professionals were undecided. Interest in learning PGx varied across the groups, with 37% of academics, 59% of clinicians, and 44% of healthcare professionals expressing interest, and 49% of academics, 28% of clinicians, and 32% of healthcare professionals being very interested (χ2=15, P=0.02, Table 3). Only 1% of scholars and 4% of healthcare professionals were uninterested, while no clinicians expressed a lack of interest (χ2=15, P=0.02). Additionally, 13% of academics, 14% of clinicians, and 20% of healthcare professionals were slightly interested (χ2=15, P=0.02). The interest analysis according to the different age groups did not show any significant association (Supplementary Table). Regarding the PGx topics of interest, the responses varied but were not significantly different. The three job positions were mainly interested in complex diseases, cancer chemotherapy, cardiovascular diseases, and diabetes mellitus (χ2=31, P=0.06, Table 3).

Ninety percent of the PhD holders affirmed their belief in PGx and its potential to improve clinical outcomes (P<0.01, Table 4). The MD participants exhibited 70% affirmative and 30% undecided responses (P<0.01). The pharmacists had the highest affirmative rate at 97%, with 3% undecided and no negative responses (P<0.01). In contrast, Bachelor-level Healthcare professionals showed 85% affirmative responses, 10% undecided, and 5% negative (P<0.01). Among PhD holders, 50% expressed a very high level of interest in learning more about pharmacogenomics (PGx), 36% reported being interested, 13% were slightly interested, and 1% indicated no interest (P<0.01). MD respondents demonstrated 60% interest, with 27% being very interested, 13% slightly interested, and no disinterest reported (P<0.01). Pharmacists showed equal proportions of being very interested and interested (42% each), with 16% slightly interested and no reports of disinterest (P<0.01). Bachelor-level healthcare professionals displayed 44% interest, 28% very interested, 22% slightly interested, and 6% not interested (P<0.01). In terms of interest in specific PGx topics, PhD respondents reported the highest levels of interest in cancer chemotherapy (27%), complex diseases (18%), and infectious diseases (13%) (χ^2=41, P=0.06). MD participants showed considerable interest in cardiovascular diseases (21%) and cancer chemotherapy (20%), while pharmacists exhibited the highest interest in these two areas (25% and 21% respectively)

(Table 4). Bachelor-level healthcare professionals showed notable interest in cancer chemotherapy (21%) and complex diseases (19%), but interest in other topics remained lower across all groups (Table 4).

Discussion

While the impact of PGx on healthcare is substantial, the realities and expectations of precision medicine vary widely across different contexts and face numerous challenges. This study is the first to investigate the current status of PGx from a specialist viewpoint on a multinational level and is the first in the Arab world to provide a comparative perspective. The results suggest higher familiarity and educational involvement with PGx in Qatar and Saudi Arabia compared to Lebanon. However, a larger proportion of respondents in Lebanon are considering integrating PGx into their practice or research activities, while more respondents in Qatar and Saudi Arabia are already doing so occasionally or routinely.

Significant differences were noted in the level of uncertainty about PGx's potential and specific topics of interest, particularly regarding diabetes mellitus (Qatar) and cancer chemotherapy (Saudi Arabia). These differences are critical for tailoring educational and implementation strategies in each country, underscoring the variability in perceptions and needs for PGx across healthcare systems with different structures. To effectively implement precision medicine in clinical practice, it is crucial to evaluate the specific context of each healthcare system, including the current state of implementation, existing obstacles, and unique challenges.

PGx implementation in research and clinical practice in Arab and Middle Eastern countries faces several documented challenges. One significant challenge is the lag in biomedical research output in these countries (45). Another is the low genomic literacy rates among healthcare professionals, leading to a gap in translating genomic knowledge to patient care (46). This disparity can hinder the development and application of PGx knowledge, necessitating concerted efforts to bridge this gap (12). PGx implementation in Lebanon mirrors challenges faced by other nearby countries. Ongoing efforts to raise awareness among healthcare professionals in Lebanon (47) indicate growing interest in this field. However, limited availability, high costs of genetic testing, and delays in translating results underscore the need for solutions to these challenges (47).

PGx challenges varied between countries, highlighting the need for tailored strategies to address specific issues. Developing comprehensive training programs for healthcare professionals, including physicians and pharmacists, can enhance their understanding and utilization of PGx in clinical decision-making (48).

Addressing economic factors in Lebanon would require policy changes to improve accessibility and affordability of PGx testing, potentially through government subsidies or insurance coverage (46, 49, 50). In Qatar, where more respondents are already integrating PGx into their practice, developing robust clinical decision support systems and consultation services could facilitate regular use of PGx in patient care.

Several recommendations can be offered based on the study's findings. Firstly, educational and implementation strategies should be tailored to each country's needs and barriers (51). In Qatar, efforts should focus on transitioning from occasional or routine PGx integration to a more comprehensive and standardized approach. In Lebanon, targeted initiatives should support and incentivize PGx integration into practice or research activities. Addressing economic factors and raising awareness about PGx's potential benefits could encourage adoption in Lebanon and other countries.

In terms of PGx interest areas, specific attention should be given to diabetes mellitus in Qatar and cancer chemotherapy in Saudi Arabia, where there is significant interest due to the high incidence of certain cancers (52-55). Breast cancer is the most commonly diagnosed cancer among Lebanese and Saudi females, with a significant increase in incidence rates over the years (54, 56). Such high incidence of breast cancer in the two countries may justify the high interest in cancer PGx across participants from Saudi Arabia (36%) and from Lebanon (27%). Our findings are in parallel with global data on PGx of cancer therapies, where the oncology field has been leading on the forefront of precision medicine (57), and almost half of the FDA approvals for pharmacogenomic testing belong to the field of cancer therapeutics (58). When examining respondents’ interest in PGx-specific topics, it becomes evident that certain diseases, such as complex diseases, cardiovascular diseases, and diabetes mellitus, may share overlapping etiology. Nevertheless, their pharmacogenomic implications and therapeutic strategies remain distinct. Internal and family medicine practitioners are often responsible for comprehensively managing complex diseases. In contrast, endocrinologists primarily address the intricate metabolic and hormonal aspects of diabetes mellitus, and cardiologists focus on the hemodynamic and structural complexities of cardiovascular diseases. By distinguishing these categories, we can capture clinicians' diverse and specialty-specific interests in PGx topics.

Collaboration with stakeholders, including healthcare professionals, educators, policymakers, and patients, is essential for successful PGx implementation (59). Engaging primary care physicians and

pharmacists can enhance their knowledge and experience with PGx testing and facilitate its integration into routine clinical practice (60). The importance of preemptive genotyping for precision medicine should be emphasized, as it can optimize medication selection and dosing, improving healthcare outcomes (61). Real-life examples of successful pharmacogenomic implementation include preemptive genotyping at institutions such as Vanderbilt and St. Jude Children's Research Hospital, which provides a basis for overcoming implementation challenges (62).

The stratified analysis according to the academic degree, showed interesting patterns (Table 4). Almost all PhD holders, pharmacists, and most MDs and bachelor-level healthcare professionals (>70%) believed that PGx could improve clinical outcomes. Interest in learning PGx varied significantly, with 50% of the PhDs being very interested, 60% of the MDs interested, and 42% and 28% of the pharmacists and bachelors (respectively) being very interested. Topic preferences were cancer chemotherapy (highest across groups), complex diseases (notable for PhDs and Bachelors), and cardiovascular diseases (preferred by MDs and Pharmacists).

Many clinicians can also have teaching activities; thus, their specialty areas might overlap with those of academics. We have carefully checked if the enrolled clinicians were also academics. Interestingly, almost all of them, except two from Lebanon, had teaching activities, and they specialize in PGx/Personalized Medicine and Medical Genetics. Interestingly, our academics had different specialties, hindering the need to analyze the data according to specialty area.

While the current results provide valuable insights into the differences between the three surveyed countries regarding familiarity, educational involvement, and challenges in integrating PGx into their healthcare systems, it is essential to highlight the limitations. First, the study's findings are based on self-reported data that can be subject to recall bias and social desirability bias, potentially influencing the accuracy of the responses. Second, the sample size and representativeness of respondents in the three countries should be considered. Third, the study may not have fully captured the diverse perspectives and experiences of healthcare professionals healthcare professionals' diverse perspectives and experiences in different practice settings, including those in rural or smaller healthcare facilities. Fourth, the survey did not gauge data on participant's awareness of existing pharmacogenomic guidelines; such data would have provided valuable insights into their familiarity with standardized practices.

This study underscores the crucial need for education and training in PGx, as the lack of awareness and expertise among healthcare professionals can pose a significant barrier to integrating PGx information into clinical decision-making processes. Arab and Middle Eastern nations face multifaceted obstacles in advancing PGx research and integrating it into clinical practice. These challenges encompass resource constraints, educational gaps, regulatory hurdles, ethical considerations, data limitations, and economic factors. A comprehensive understanding of these challenges is crucial for devising effective strategies to overcome them.

References:

1. Lander ES, Linton LM, Birren B, Nusbaum C, Zody MC, Baldwin J, et al. Initial sequencing and analysis of the human genome. Nature. 2001;409(6822):860-921.
2. Dickmann LJ, Ware JA. Pharmacogenomics in the age of personalized medicine. Drug Discov Today Technol. 2016;21-22:11-6.
3. Singh DB. The Impact of Pharmacogenomics in Personalized Medicine. Adv Biochem Eng Biotechnol. 2020;171:369-94.
4. What is the difference between precision medicine and personalized medicine? What about pharmacogenomics? [Available from: https://medlineplus.gov/genetics/understanding/precisionmedicine/precisionvspersonalized/.
5. Delpierre C, Lefèvre T. Precision and personalized medicine: What their current definition says and silences about the model of health they promote. Implication for the development of personalized health. Front Sociol. 2023;8:1112159.
6. Sadee W, Wang D, Hartmann K, Toland AE. Pharmacogenomics: Driving Personalized Medicine. Pharmacol Rev. 2023;75(4):789-814.
7. US Food and Drug Administration. Table of valid genomic biomarkers in the context of approved drug labels 2024 [Available from: https://www.fda.gov/drugs/science-and-research-drugs/table-pharmacogenomic-biomarkers-drug-labeling.
8. Pirmohamed M. Pharmacogenomics: current status and future perspectives. Nat Rev Genet. 2023;24(6):350-62.
9. van der Lee M, Kriek M, Guchelaar HJ, Swen JJ. Technologies for Pharmacogenomics: A Review. Genes (Basel). 2020;11(12).
10. Sukri A, Salleh MZ, Masimirembwa C, Teh LK. A systematic review on the cost effectiveness of pharmacogenomics in developing countries: implementation challenges. Pharmacogenomics J. 2022;22(3):147-59.
11. Klein ME, Parvez MM, Shin JG. Clinical Implementation of Pharmacogenomics for Personalized Precision Medicine: Barriers and Solutions. J Pharm Sci. 2017;106(9):2368-79.
12. El Shamieh S, Zgheib NK. Pharmacogenetics in developing countries and low resource environments. Hum Genet. 2022;141(6):1159-64.
13. Zgheib NK, Patrinos GP, Akika R, Mahfouz R. Precision Medicine in Low- and Middle-Income Countries. Clin Pharmacol Ther. 2020;107(1):29-32.
14. Ayati N, Afzali M, Hasanzad M, Kebriaeezadeh A, Rajabzadeh A, Nikfar S. Pharmacogenomics Implementation and Hurdles to Overcome; In the Context of a Developing Country. Iran J Pharm Res. 2021;20(4):92-106.
15. Gameiro GR, Sinkunas V, Liguori GR, Auler-Júnior JOC. Precision Medicine: Changing the way we think about healthcare. Clinics (Sao Paulo). 2018;73:e723.
16. Zgheib NK. The Pharmacogenetics Laboratory of the Department of Pharmacology and Toxicology at the American University of Beirut Faculty of Medicine. Pharmacogenomics. 2017;18(14):1311-6.
17. Awada Z, Haider S, Tfayli A, Bazarbachi A, El-Saghir NS, Salem Z, et al. Pharmacogenomics variation in drug metabolizing enzymes and transporters in relation to docetaxel toxicity in Lebanese breast cancer patients: paving the way for OMICs in low and middle income countries. Omics. 2013;17(7):353-67.
18. Maamari D, El-Khoury H, Saifi O, Muwakkit SA, Zgheib NK. Implementation of Pharmacogenetics to Individualize Treatment Regimens for Children with Acute Lymphoblastic Leukemia. Pharmgenomics Pers Med. 2020;13:295-317.

19. Zgheib NK, Branch RA. Drug metabolism and liver disease: a drug-gene-environment interaction. Drug Metab Rev. 2017;49(1):35-55.
20. Zgheib NK, Alameddine R, Massoud R, Nasr R, Zahreddine A, El Cheikh J, et al. The role of candidate genetic polymorphisms in the interaction between voriconazole and cyclosporine in patients undergoing allogeneic hematopoietic cell transplantation: An explorative study. Curr Res Transl Med. 2020;68(2):51-8.
21. Saad H, Jabotian K, Sakr C, Mahfouz R, Akl IB, Zgheib NK. The Role of Angiotensin Converting Enzyme 1 Insertion/Deletion Genetic Polymorphism in the Risk and Severity of COVID-19 Infection. Front Med (Lausanne). 2021;8:798571.
22. Naja K, El Shamieh S, Fakhoury R. rs622342A>C in SLC22A1 is associated with metformin pharmacokinetics and glycemic response. Drug Metab Pharmacokinet. 2020;35(1):160-4.
23. Naja K, Salami A, El Shamieh S, Fakhoury R. rs622342 in SLC22A1, CYP2C9*2 and CYP2C9*3 and Glycemic Response in Individuals with Type 2 Diabetes Mellitus Receiving Metformin/Sulfonylurea Combination Therapy: 6-Month Follow-Up Study. J Pers Med. 2020;10(2).
24. Zgheib N, Ghaddar F, Sabra R. Teaching Pharmacogenetics on Low and Middle-Income Countries: Team Based Learning and Lessons Learned at The American University Of Beirut. Current Pharmacogenomics and Personalized Medicine. 2011;9:25-40.
25. Digihealth project: Elevating and Enriching a Pharmacogenomics course Through the Use of Open Education Resources 2021 [Available from: https://digihealth.uni-med.net/elevating-and-enriching-a-pharmacogenomics-course-through-the-use-of-open-education-resources/".
26. Zgheib NK, Arawi T, Mahfouz RA, Sabra R. Attitudes of health care professionals toward pharmacogenetic testing. Mol Diagn Ther. 2011;15(2):115-22.
27. Mehtar M, Hammoud SH, Amin MEK. An objective evaluation of fundamental pharmacogenomics knowledge among pharmacists and pharmacy students. Saudi Pharm J. 2022;30(12):1765-72.
28. Abou Diwan E, Zeitoun RI, Abou Haidar L, Cascorbi I, Khoueiry Zgheib N. Implementation and obstacles of pharmacogenetics in clinical practice: An international survey. Br J Clin Pharmacol. 2019;85(9):2076-88.
29. Bastaki K, Velayutham D, Irfan A, Adnan M, Mohammed S, Mbarek H, et al. Forging the path to precision medicine in Qatar: a public health perspective on pharmacogenomics initiatives. Front Public Health. 2024;12:1364221.
30. Hendi NN, Al-Sarraj Y, Ismail Umlai UK, Suhre K, Nemer G, Albagha O. Genetic determinants of Vitamin D deficiency in the Middle Eastern Qatari population: a genome-wide association study. Front Nutr. 2023;10:1242257.
31. Velayutham D, Bastaki K, Ghuloum S, Azeem MW, Pirmohamed M, Jithesh PV. Clinically actionable pharmacogenomic landscape of antidepressants and antipsychotics in Qatar: A population-based cohort study. medRxiv. 2023:2023.09.27.23296201.
32. Bastaki K, El Anbari M, Ghuloum S, Jithesh PV. Prescription Pattern of Antidepressants and the Potential for Personalized Medicine in the Qatari Population. J Pers Med. 2021;11(5).
33. Jithesh PV, Abuhaliqa M, Syed N, Ahmed I, El Anbari M, Bastaki K, et al. A population study of clinically actionable genetic variation affecting drug response from the Middle East. NPJ Genom Med. 2022;7(1):10.
34. Ahmed I, Ziab M, Mbarak H, Taheri S, Chagoury O, Hussain SA, et al. Improved Type 2 Diabetes Risk Stratification in the Qatar Biobank Cohort by Ensemble Learning Classifier Incorporating Multi-Trait, Population-Specific, Polygenic Risk Scores. medRxiv. 2023:2023.06.23.23291830.
35. Qatar Genome publications [Available from: https://www.qatargenome.org.qa/research/publications.

36. Elewa H, Alkhiyami D, Alsahan D, Abdel-Aziz A. A survey on the awareness and attitude of pharmacists and doctors towards the application of pharmacogenomics and its challenges in Qatar. J Eval Clin Pract. 2015;21(4):703-9.
37. Alrabiah Z, Syed W, Babelghaith SD, Al Arifi MN. Clinical Knowledge, Attitude, and Perceptions of Community Pharmacists Towards Pharmacogenomics - A Cross-Sectional Study from Saudi Arabia. Pharmgenomics Pers Med. 2023;16:433-41.
38. Mensah GA, Fuster V, Murray CJL, Roth GA. Global Burden of Cardiovascular Diseases and Risks, 1990-2022. J Am Coll Cardiol. 2023;82(25):2350-473.
39. Goljan E, Abouelhoda M, Tahir A, ElKalioby M, Meyer B, Monies D. Large-scale next generation sequencing based analysis of SLCO1B1 pharmacogenetics variants in the Saudi population. Hum Genomics. 2024;18(1):30.
40. Alhazzani AA, Munisamy M, Karunakaran G. Pharmacogenetics of CYP2C19 genetic polymorphism on clopidogrel response in patients with ischemic stroke from Saudi Arabia. Neurosciences (Riyadh). 2017;22(1):31-7.
41. Swen JJ, Nijenhuis M, van Rhenen M, de Boer-Veger NJ, Buunk AM, Houwink EJF, et al. Pharmacogenetic Information in Clinical Guidelines: The European Perspective. Clin Pharmacol Ther. 2018;103(5):795-801.
42. Algahtani M. Knowledge, Perception, and Application of Pharmacogenomics Among Hospital Pharmacists in Saudi Arabia. Risk Manag Healthc Policy. 2020;13:1279-91.
43. Cuschieri S. The STROBE guidelines. Saudi J Anaesth. 2019;13(Suppl 1):S31-s4.
44. Ghaddar F, Cascorbi I, Zgheib NK. Clinical implementation of pharmacogenetics: a nonrepresentative explorative survey to participants of WorldPharma 2010. Pharmacogenomics. 2011;12(7):1051-9.
45. Benamer HT, Bakoush O. Arab nations lagging behind other Middle Eastern countries in biomedical research: a comparative study. BMC Med Res Methodol. 2009;9:26.
46. Hippman C, Nislow C. Pharmacogenomic Testing: Clinical Evidence and Implementation Challenges. J Pers Med. 2019;9(3).
47. Ossaily S, Zgheib NK. The pharmacogenetics of drug metabolizing enzymes in the Lebanese population. Drug Metabol Drug Interact. 2014;29(2):81-90.
48. Johansen Taber KA, Dickinson BD. Pharmacogenomic knowledge gaps and educational resource needs among physicians in selected specialties. Pharmgenomics Pers Med. 2014;7:145-62.
49. The World Bank In Lebanon [updated Nov 02, 2022. Available from: https://www.worldbank.org/en/country/lebanon/overview.
50. Bou Sanayeh E, El Chamieh C. The fragile healthcare system in Lebanon: sounding the alarm about its possible collapse. Health Econ Rev. 2023;13(1):21.
51. Roman YM. Editorial: The role of pharmacogenomics in addressing health disparities: the path, the promise, and the barriers. Front Genet. 2023;14:1233045.
52. Qattan MY. The Incidence Rate of Non-Hodgkin's Lymphoma in Saudi Arabia: An Observational Descriptive Population Based Epidemiological Study from the Saudi Cancer Registry (2006–2016). Open Access Macedonian Journal of Medical Sciences. 2023;11(B):445-52.
53. Alsalem A, Alessa M, Alshaalan Y, Alkahtani N, Alamoudi M. Trends in the Prevalence and Incidence of Thyroid Cancer in Saudi Arabia Over the Last Decade: A Retrospective Cohort Study of 9882 Patients. 2023.
54. Basudan AM. Breast Cancer Incidence Patterns in the Saudi Female Population: A 17-Year Retrospective Analysis. Medicina (Kaunas). 2022;58(11).
55. Alghamdi IG. Epidemiology of gastric cancer in Saudi Arabia from 2004 to 2017. Mol Clin Oncol. 2023;19(5):93.

56. Fares MY, Salhab HA, Khachfe HH, Khachfe HM. Breast Cancer Epidemiology among Lebanese Women: An 11-Year Analysis. Medicina (Kaunas). 2019;55(8).
57. Saadeh C, Bright D, Rustem D. Precision Medicine in Oncology Pharmacy Practice. Acta Med Acad. 2019;48(1):90-104.
58. Kim JA, Ceccarelli R, Lu CY. Pharmacogenomic Biomarkers in US FDA-Approved Drug Labels (2000-2020). J Pers Med. 2021;11(3).
59. Bank PCD, Swen JJ, Guchelaar HJ. Implementation of Pharmacogenomics in Everyday Clinical Settings. Adv Pharmacol. 2018;83:219-46.
60. Haga SB. The Critical Role of Pharmacists in the Clinical Delivery of Pharmacogenetics in the U.S. Pharmacy (Basel). 2023;11(5).
61. Gallaway KA, Cann K, Oetting K, Rothenberger M, Raibulet A, Slaven JE, et al. The Potential Impact of Preemptive Pharmacogenetic Genotyping in the Neonatal Intensive Care Unit. J Pediatr. 2023;259:113489.
62. Rafi I, Crinson I, Dawes M, Rafi D, Pirmohamed M, Walter FM. The implementation of pharmacogenomics into UK general practice: a qualitative study exploring barriers, challenges and opportunities. J Community Genet. 2020;11(3):269-77.

Table 1: Demographic characteristics of the study participants in Lebanon, Qatar, and Saudi Arabia.

**Demographic characteristics**	**All (N=337)**		**Lebanon (N=145)**		**Qatar (N=137)**		**Saudi Arabia (N=55)**	
**Age categories N(%)**								
18-25	103	(30%)	95	(66%)	6	(4.5%)	2	(4%)
26-35	84	(25%)	26	(18%)	45	(33%)	13	(24%)
36-45	105	(32%)	15	(10%)	58	(42%)	32	(57%)
46-55	35	(10%)	6	(4%)	22	(16%)	7	(13%)
> 55	10	(3%)	3	(2%)	6	(4.5%)	1	(2%)
**Gender N(%)**								
Male	118	(35%)	49	(34%)	41	(30%)	28	(51%)
Female	219	(65%)	96	(66%)	96	(70%)	27	(49%)
**Job N(%)**								
Academic	106	(32%)	33	(23%)	34	(25%)	39	(70%)
Clinician	29	(9%)	9	(6%)	13	(9%)	8	(15%)
Healthcare Professional	202	(59%)	103	(71%)	90	(66%)	8	(15%)
Academic Degree N(%)								
PhD	101	(30%)	29	(20%)	34	(25%)	38	(69%)
MD	30	(9%)	10	(7%)	13	(9%)	7	(13%)
BS in Pharmacy	64	(19%)	9	(6%)	45	(33%)	10	(18%)
Bachelor	142	(42%)	97	(67%)	45	(33%)	0	
**Professional Experience N(%)**								
< 1 year	62	(18%)	39	(27%)	17	(12%)	6	(11%)
1-5 years	115	(34%)	72	(50%)	34	(25%)	9	(16%)
6-10 years	57	(17%)	12	(8%)	27	(20%)	18	(33%)
11-20 years	74	(22%)	17	(12%)	42	(31%)	15	(27%)
> 20 years	29	(9%)	5	(3%)	17	(12%)	7	(13%)

For continuous variables, values are the arithmetic mean ± SD. Categorical variables are shown as numbers (n) and percentages (%).
n: sample size.

Table 2: Future directions and interest in pharmacogenomics in Lebanon, Qatar, and Saudi Arabia.

	Lebanon		Qatar		Saudi Arabia		χ2	P
**Do you believe that pharmacogenomics has the potential to significantly improve patient outcomes in clinical practice?**								
No	6	(4%)	3	(2%)	0			
Yes	125	(86%)	117	(86%)	52	(94%)	5	0.278
Undecided	14	(10%)	17	(12%)	3	(6%)		
**How interested would you be in learning about pharmacogenomics?**								
Not Interested	3	(2%)	7	(5%)	0			
Slightly Interested	27	(19%)	29	(21%)	2	(4%)		
Interested	61	(42%)	61	(45%)	23	(42%)	18	0.005
Very Interested	54	(37%)	40	(29%)	30	(55%)		
**What specific pharmacogenomics topics, if any, would you like to learn about?**								
Cancer chemotherapy	39	(27%)	21	(15%)	20	(36%)		
Cardiovascular diseases	18	(12%)	27	(20%)	10	(18%)		
Complex diseases*	41	(28%)	4	(3%)	12	(22%)		
Diabetes Mellitus	0		34	(25%)	0			
Immune Suppressants	0		9	(7%)	0			
Infectious diseases	15	(10%)	11	(8%)	4	(7%)6	141	<0.001
Neurodegenerative disorders	8	(6%)	9	(6%)	7	(13%)		
Pain management	0		12	(9%)	0			
Precision vaccines and immunity	8	(6%)	0		0			
Psychiatric disorders	12	(8%)	10	(7%)	0			
Rare diseases	4	(3%)	0	0	2	(4%)		

* Complex diseases are common diseases caused by a combination of genetic, environmental, and lifestyle factors.

For continuous variables, values are the arithmetic mean ± SD.

Categorical variables are shown as numbers (n) and percentages (%).n: sample size. A χ2 test of independence was used to test if the differences between Lebanon and water are significantly different.

Table 3: Future directions and Interest in pharmacogenomics according to participants' jobs.

	Academic		Clinician		Healthcare professional		χ2	P
**Do you believe that pharmacogenomics has the potential to significantly improve patient outcomes in clinical practice?**								
No	1	(1%)	0		8	(4%)		
Undecided	9	(8%)	9	(31%)	16	(8%)	19	0.001
Yes	96	(91%)	20	(69%)	178	(88%)		
**How interested would you be in learning about pharmacogenomics?**								
Interested	39	(37%)	17	(59%)	89	(44%)		
Not interested	1	(1%)	0	0	9	(4%)	15	0.02
Slightly	14	(13%)	4	(14%)	40	(20%)		
Very Interested	52	(49%)	8	(28%)	64	(32%)		
**What specific pharmacogenomics topics, if any, would you like to learn about?**								
All the complex diseases*	19	(18%)	5	(17%)	33	(16%)		
Cancer chemotherapy	29	(27%)	6	(21%)	45	(22%)		
Cardiovascular diseases	13	(12%)	6	(21%)	36	(18%)		
Diabetes Mellitus	5	(5%)	4	(14%)	25	(12%)		
Immune Suppressants	2	(2%)	1	(3%)	6	(3%)		
Infectious diseases	14	(13%)	3	(10%)	13	(6%)	31	0.06
Neurodegenerative disorders	12	(11%)	0		12	(6%)		
Pain management	2	(2%)	0		10	(5%)		
Personalized vaccines and immunity	4	(4%)	0		4	(2%)		
Psychiatric disorders	3	(3%)	2	(7%)	17	(8%)		
Rare diseases	3	(3%)	2	(7%)	1	(0%)		

* Complex diseases are common diseases caused by a combination of genetic, environmental, and lifestyle factors.

For continuous variables, values are the arithmetic mean ± SD. Categorical variables are shown as numbers (n) and percentages (%). n: sample size. A χ2 test of independence was used to test if the differences between Lebanon and water are significantly different.

Table 4: Future directions and Interest in pharmacogenomics according to the academic Degree.

	PhD		MD		Bachelor in Pharmacy		Bachelor		χ2	P
No	1	(1%)			0		8	(5%)		
Undecided	9	(9%)	9	(30%)	2	(3%)	14	(10%)	25	<0.01
Yes	91	(90%)	21	(70%)	62	(97%)	120	(85%)		
Interested	37	(36%)	18	(60%)	27	(42%)	63	(44%)		
Not interested	1	(1%)			0		9	(6%)		<0.01
Slightly	13	(13%)	4	(13%)	10	(16%)	31	(22%)	24	
Very Interested	50	(50%)	8	(27%)	27	(42%)	39	(28%)		
All the complex diseases*	18	(18%)	5	(17%)	5	(8%)	29	(19%)		
Cancer chemotherapy	28	(27%)	6	(20%)	15	(21%)	32	(21%)		
Cardiovascular diseases	13	(13%)	6	(21%)	16	(25%)	20	(14%)		
Diabetes Mellitus	5	(5%)	4	(13%)	4	(6%)	21	(14%)		
Immune Suppressants	2	(2%)	1	(3%)	3	(5%)	3	(2%)		
Infectious diseases	13	(13%)	3	(10%)	4	(6%)	10	(5%)		
Neurodegenerative disorders	11	(11%)	1	(3%)	6	(10%)	6	(3%)	4S1	0.06
Pain management	2	(2%)			5	(8%)	5	(2%)		
Personalized vaccines and immunity	3	(3%)			2	(3%)	3	(12%)		
Psychiatric disorders	3	(3%)	2	(7%)	5	(8%)	12	(7%)		
Rare diseases	3	(3%)	2	(7%)	0		1	(1%)		

* Complex diseases are common diseases caused by a combination of genetic, environmental, and lifestyle factors.

For continuous variables, values are the arithmetic mean ± SD. Categorical variables are shown as numbers (n) and percentages (%). n: sample size. A χ2 test of independence was used to test if the differences between Lebanon and water are significantly different.

Figure 1: Knowledge and attitudes towards implementation of pharmacogenomics in clinical practice and research.

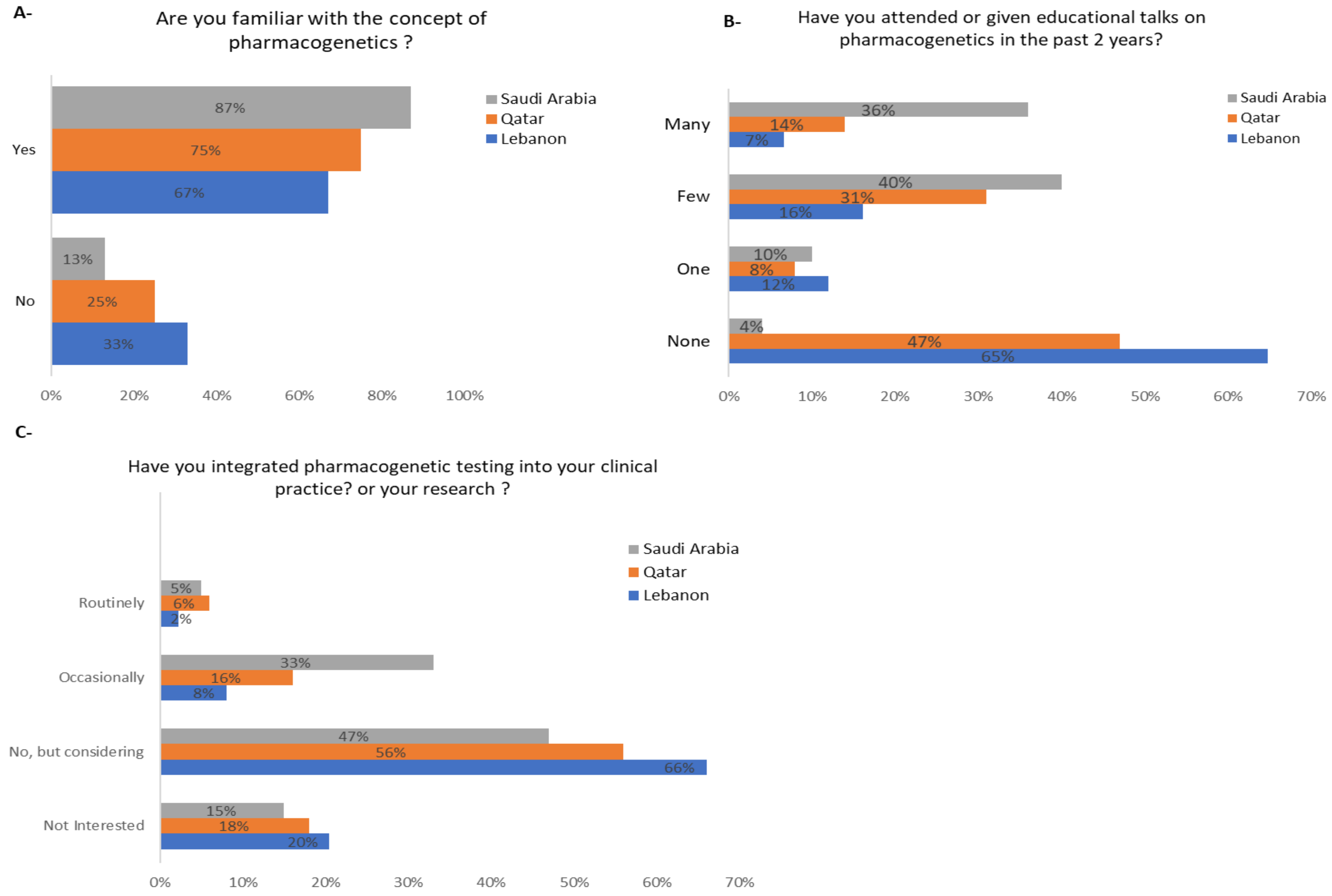

A: Familiarity with the Concept of Pharmacogenomics. B. Attendance or Presentation of Educational Talks on Pharmacogenomics in the Past 2 Years. C. Integration of Pharmacogenomics into Clinical Practice or Research Activities.

Figure 2: The overall Challenges for bringing pharmacogenetics into clinical practice.

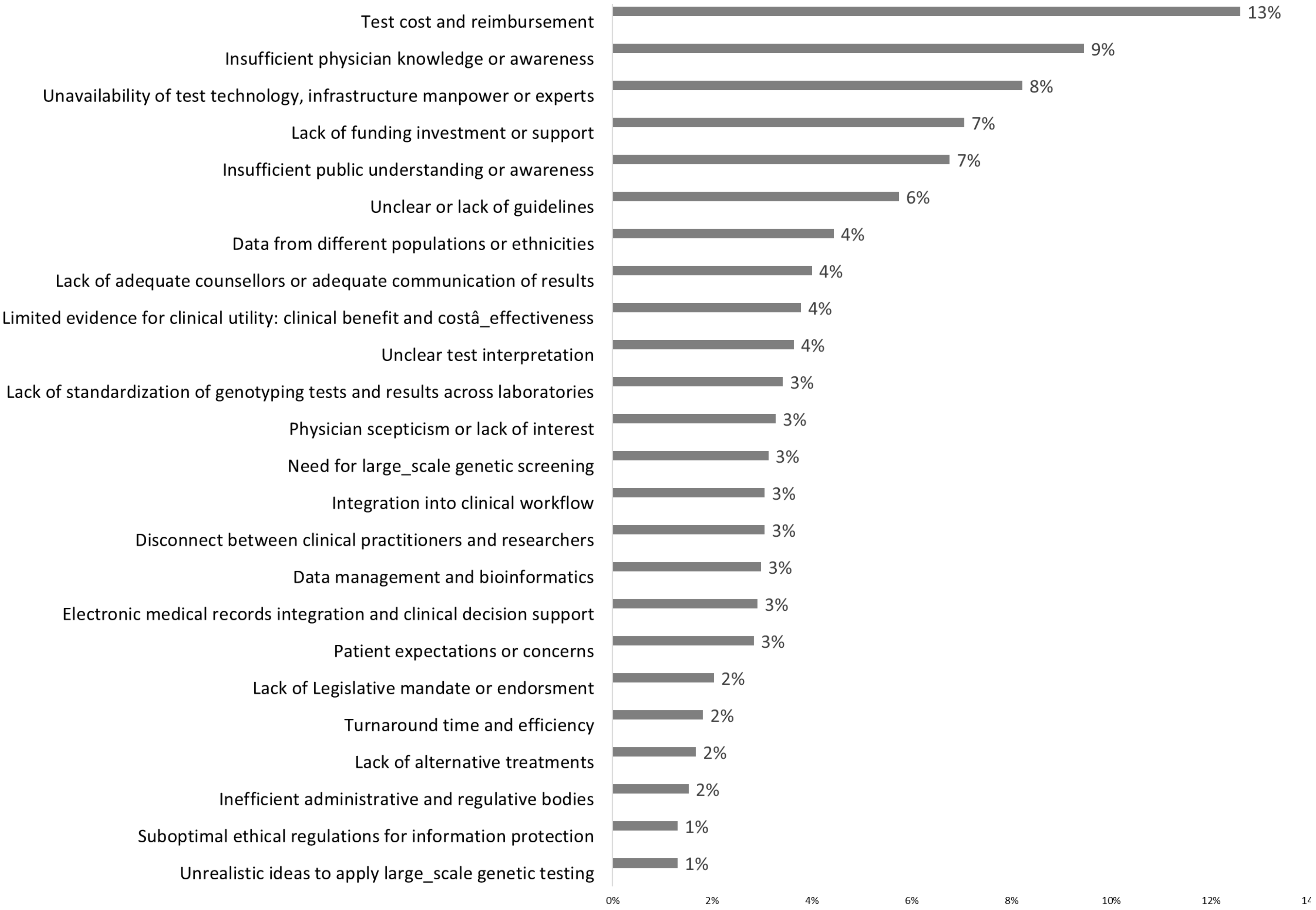

Figure 3: Challenges for bringing pharmacogenetics into clinical practice in Lebanon, Qatar, and Saudi Arabia.

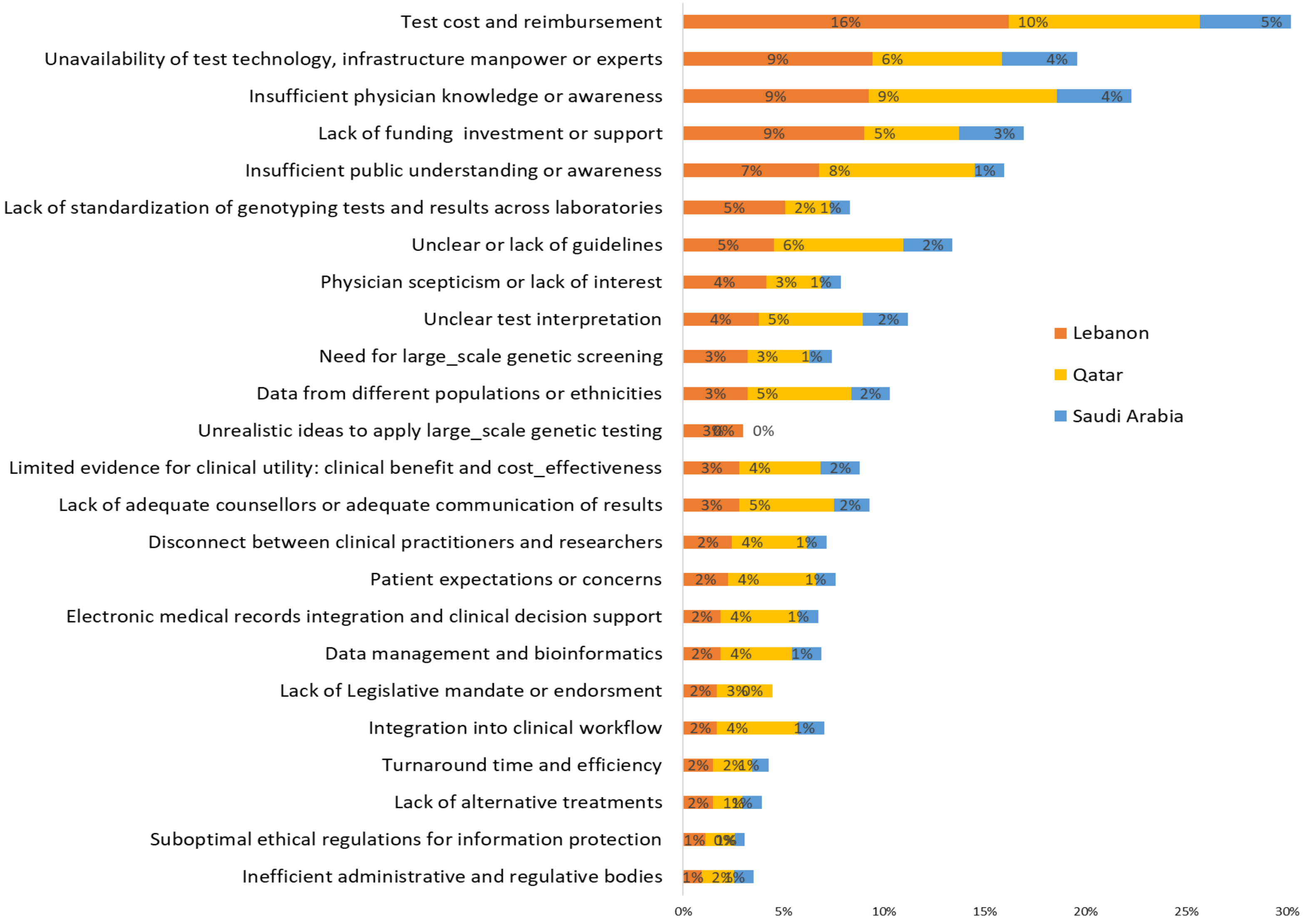

Reviewer #1:

1. Do you have the data on the level of education for the subjects? (eg. PhD, Masters, Bsc) would be interesting to see if there is a difference in acceptance towards Pgx between each group

We thank the reviewer for the pertinent comment. Accordingly, we have added all our participants' academic degrees (Table 1, page 20). We have described these results (lines 177-182, page 8) and then compared the difference in acceptance towards PGx in each group (Table 4, page 23).

This analysis showed interesting patterns that we describe in the Results section (lines 247-261, page 10 and lines 262-263, page 11) and the Discussion section (line 336-342, page 14).

2. Would be great if you also have data on their awareness of any guidelines related to Pgx.

We thank the reviewer for this important comment. Unfortunately, we don't have this data and we have added this to the limitations section (line 357-359, page 14)

3. Could you elaborate more on the level of interest (for groups who are interested to know more) with age?

We thank the reviewer for highlighting this issue. We analyzed the interest level according to the different age groups and found no significant associations (Supplementary Table). We also describe this in the results section (lines 241-242, page 10).

4. Is there data on pgx practice in Lebanon, Qatar, and Saudi Arabia, and does it collaborate with the level of interest for each respondent grouped by countries?

We thank the reviewer for this comment. Data on pharmacogenomics practice in the three countries exists to some extent in literature, and this was highlighted in the Introduction section with relevant references: references 16-27 for Lebanon, 29-36 for Qatar, and 37-40 for Saudi Arabia. Yes, the level of interest varied across the three countries, as shown in Table 2 (page 21). We have described all the details in the results section (lines 186-196, page 8).

Reviewer #2: Reviewer Comments:

1. Title Appropriateness: The title of the manuscript is not suitable as it refers to the Middle East, which comprises 18 countries. However, the study is limited to three countries: Lebanon, Qatar, and Saudi Arabia. The data presented may not adequately represent the healthcare population of the entire region. I recommend revising the title to "Integrating Pharmacogenomics in Three Middle Eastern Countries' Healthcare (Lebanon, Qatar, and Saudi Arabia): Current Insights, Challenges, and Strategic Direction" to more accurately reflect the scope of the research.

We thank the reviewer for pointing out this issue. We have used the reviewer's suggestion for the title of our article.

2. Data Sufficiency: The results and areas of coverage presented in the manuscript are insufficient for publication in a Q1 journal. To enhance the study's contribution to the field, I suggest providing a broader dataset or deeper insights into the healthcare practices and pharmacogenomics landscape within the selected countries.

We thank the reviewer for being keen about data coverage presented in the manuscript. Indeed, a broader dataset would be beneficial suggestion, and providing it would be interesting for ongoing research. For the current study, we exhausted different means of contacting pharmacogenomics experts in the three countries, by approaching them personally, given their known expertise in the field and their reputation. We also sought their support in disseminating the survey among their teams. We reached to broader pharmacist communities to seek specialist participation. Given that the primary authors from each country are academicians, we used our contacts among specialized academicians and researchers to get more engagement in the survey. However, we need to appreciate the relative novelty of the field in the region and the fact that Qatar and Lebanon are both small countries. Finding specialists within the pharmacogenomics domain whose participation adds value to the study should be endeavored carefully. Nevertheless, we have accounted for sample size as one of the study limitations in the Discussion section.

To satisfy the reviewer's concerns and enhance the study contribution to the field, we provided deeper insights into our data by additional analysis on academic degrees, interests in pharmacogenomics, and age. The analysis according to the academic degree showed significantly different patterns as the interest for PGX varied depending on the academic degree (PhD vs. MD vs. BS in Pharmacy vs. BS in a healthcare field (Results, lines 247-261, page 10 and lines 262-263, page 11, and Table 4, page 23). In contrast, the age of the participants was not associated with the interest levels (lines 241-242, page 10).

3. Clarification of Roles: The roles of academics, healthcare professionals, and clinicians appear to overlap without clear distinctions. The manuscript should specify the areas of specialty for the academic participants, as both clinicians and healthcare professionals may also serve as academicians. Clarifying these roles will strengthen the manuscript's clarity and precision.

We thank the reviewer for pointing out this pertinent issue. After carefully checking the clinicians enrolled in our study, we noticed that only two (from Lebanon) had teaching activities. The latter

clinicians specialized in Pharmacogenomics/Personalized Medicine and Medical Genetics. Interestingly, our academics were all those of these two specialties. As this is a critical point, we have now clarified it in the Discussion section (page 14, line 344-348).

4. Statistical Analysis Issues: The statistical analysis presented is inadequate. For example, the statement, "A χ2 test of independence was used to test if the differences between Lebanon and water are significantly different," is confusing and lacks clarity. It does not provide a significance value for the measurable variables, which is essential for interpreting the results. I recommend revising this section to include clear explanations of the statistical methods used and the significance of the findings.

We do entirely agree with the reviewer and acknowledge that we didn't provide initially enough details concerning the statistical analysis section. Accordingly, we have provided all the necessary details in the methods section (lines 142-157, page 6).

5. Overlap in Pharmacogenomic Specifications: There are overlapping specifications regarding pharmacogenomics topics. For instance, conditions such as diabetes mellitus and cardiovascular diseases may be influenced by a combination of genetic, environmental, and lifestyle factors, which are similar to the characteristics of complex diseases. It is important to delineate these topics clearly to avoid confusion and enhance the manuscript's focus.

We agree with the reviewer's observation regarding the overlap in some pharmacogenomics topics. In response, we emphasize that including these overlapping categories was a deliberate decision to reflect our respondents' varied interests and areas of expertise. Although these conditions share a common etiology, their pharmacogenomic implications and therapeutic approaches are distinct. For instance, internal medicine and family medicine practitioners typically manage complex diseases. In contrast, endocrinologists predominantly focus on endocrine disorders like diabetes mellitus, while cardiologists specialize in cardiovascular diseases. By delineating these categories, we captured clinicians' variable interest in PGx topics. We have now discussed this point (lines 318-325, page 13).

6. Discussion Quality: The discussion section is well-written and effectively outlines the challenges faced in the integration of pharmacogenomics, along with proposed strategies to overcome these obstacles. This part of the manuscript is commendable and adds significant value to the overall narrative.

We thank the reviewers for the interest they showed in recommending our discussion section and highlighting its value.

Based on the above, we hope to have satisfied the reviewers' comments and to have the amended version of the manuscript acceptable for publication in PLOS One.
